# Survival factor SvfA plays multiple roles in differentiation and is essential for completion of sexual development in *Aspergillus nidulans*

**DOI:** 10.1038/s41598-020-62455-4

**Published:** 2020-03-27

**Authors:** Joo-Yeon Lim, Eun-Hye Kang, Yun-Hee Park, Jun-Ho Kook, Hee-Moon Park

**Affiliations:** Department of Microbiology and Molecular Biology, College of Bioscience and Biotechnology, Chungnam National University, Daejeon, 34134 Korea

**Keywords:** Fungal genetics, Fungal genetics

## Abstract

The first member of the *velvet* family of proteins, VeA, regulates sexual development and secondary metabolism in the filamentous fungus *Aspergillus nidulans*. In our study, through comparative proteome analysis using wild type and *veA-*deletion strains, new putative regulators of sexual development were identified and functionally analyzed. Among these, SvfA, containing a yeast survival factor 1 domain, plays multiple roles in the growth and differentiation of *A*. *nidulans*. Deletion of the *svfA* gene resulted in increased sensitivity to oxidative and cold stress as in yeast. The *svfA-*deletion strain showed an increase in bi-polar germination and a decrease in radial growth rate. The deletion strain formed structurally abnormal conidiophores and thus produced lower amounts of conidiospores during asexual development. The *svfA-*deletion strain produced few Hülle cells and small cleistothecia with no ascospores, indicating the requirement of *svfA* for the completion of sexual development. Transcription and genetic analyses indicated that SvfA modulates the expression of key development regulatory genes. Western blot analysis revealed two forms of SvfA. The larger form showed sexual-specific and VeA-dependent production. Also, the deletion of *svfA* caused decreased ST (sterigmatocystin) production. We propose that SvfA is a novel central regulator of growth, differentiation and secondary metabolism in *A. nidulans*.

## Introduction

*Aspergillus nidulans* is a model filamentous fungus that belongs to the phylum Ascomycota. Both asexual and sexual cycles allow the study of various cellular events such as development, stress responses, and secondary metabolism^1–4^. Asexual development proceeds in multiple steps with special organs: a foot cell, stalk, vesicle, metulae, phialides, and conidiospores^5^. While the vegetative cell goes through asexual development under light conditions, sexual development is favored under dark and hypoxic conditions^6^. In the sexual reproductive organ, cleistothecium, numerous asci containing eight red-purple ascospores are developed^1,7,8^.

The *velvet* family proteins, including VeA, VelB, VelC, and VosA, comprise highly conserved fungal specific regulators in ascomycetes and basidiomycetes^9^. This superfamily plays critical roles in development and secondary metabolism by forming complexes with multiple interacting partners^4,10–12^. VeA, the first identified protein in this family, controls the asexual or sexual development in response to external signals such as light and air. This regulatory function of VeA is dependent on its localization. Under light conditions, VeA disperses in both the cytoplasm and the nuclei. Under dark conditions, the VelB-VeA dimer enters the nucleus with the help of importin α (KapA), and forms a trimeric complex with the global regulator of secondary metabolism, LaeA^4,11,13–15^. Sexual development and secondary metabolism are induced by the VelB-VeA-LaeA complex and VelC^12^. Asexual development is inhibited by the VosA homodimer^9^. However, the presence of light blocks the entry of VelB-VeA dimer into the nucleus. One of the LaeA-like methyltransferases, LlmF, interacts with VeA in the cytoplasm, which causes VelB to interact with another VelB or VosA but not VeA. The accumulation of VelB-VelB and VelB-VosA complexes in the nucleus positively regulates asexual development^4,16^.

While strains with *veA1* point mutation produce more conidia and fewer cleistothecia than the wild type (WT), the *veA*-deletion strain (Δ*veA*) fails to produce cleistothecia even under dark conditions^17,18^. Expression of genes such as *aflR* and *stcU*, which are required for the biosynthesis of sterigmatocystin (ST, a precursor of aflatoxin), is decreased in the Δ*veA* strain^19^.

Other direct interacting partners of VeA are *velvet* interacting proteins (Vips). VipC, a methyltransferase, is required for repression of sexual development and can interact with a membrane protein, VapA, and another methyltransferase, VapB. VipC-VapB dimers are detached from the VapA-VipC-VapB complex at the plasma membrane by external signals and prevent VeA from entering the nucleus^20^.

Although the *velvet* family and interactors are known to be involved in sexual development, further studies are needed to find novel factors and to understand the molecular process of sexual development in *A. nidulans*. Here, we report the results from comparative proteome analyses of WT and Δ*veA* strains to identify novel VeA-dependent proteins (Vdps), the expression of which could be affected by the absence of VeA during development. Among the 144 proteins identified, four Vdps showing significant reductions in their intensity during the sexual stage in the Δ*veA* strain were analyzed by gene-deletion experiments and functional assays. In this report, we show that SvfA, a homolog of yeast survival factor 1, is required for response to oxidative- and cold-stress, and is a novel regulator that plays multiple roles in the regulation of growth and differentiation, which is essential for completion of sexual development in *A. nidulans*.

## Results

### Detection and identification of proteins affected by VeA

**Figure 1:**
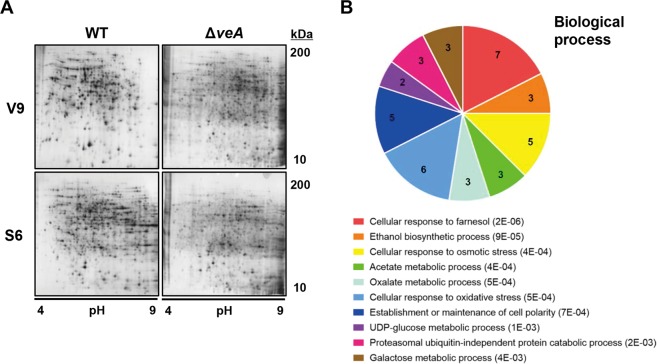
Proteome analysis of WT and Δ*veA* strains. (**A**) A representative map of 2-DE analysis from three independent replicates. Equal amounts of total proteins at the vegetative stage (V9; 9 h) and sexual stage (S6; 6 h) were separated by 2-DE and visualized with silver staining. (**B**) Gene ontology (GO) enrichment analysis of down-regulated proteins in the Δ*veA* strains. In each case, the top 10 significantly enriched biological processes are shown along with *p*-value computed using the Benjamini-Hochberg procedure^54^. The GO enrichment analysis was performed using FungiFun2 webserver^55^.

VeA regulates development and secondary metabolism in *A. nidulans* through its interactions with other regulatory proteins, including VelB, VosA, LaeA, and Vips, and through feedback control on the expression of various genes^15,20^. For screening the VeA-target proteins, mycelial balls of *veA-*deletion strain (Δ*veA*) and wild type strain (*veA*^+^), grown in liquid complete medium, were transferred to agar plates to induce sexual development^21^. Whole-cell lysates from these samples, harvested at different developmental stages, were analyzed on 2-DE^22,23^ in triplicates, with independently harvested samples (Supplementary Fig. S1). About 2,400 protein spots were detected on the 2-DE gels (Fig. 1A). By comparing the gel profiles of *veA*^+^ and Δ*veA* strains at the same stage, spots showing significant changes in their intensity were selected and subjected to in-gel tryptic digestion and MALDI-TOF. Among the 200 spots analyzed, only 144 proteins were identified. Out of the 76 protein that were down-regulated during the sexual stage in the Δ*veA* strain, 56 were significantly enriched in the FunCat categories (P ≤ 0.05). The top 10 enriched FunCat categories were as follows: cellular response to farnesol, ethanol biosynthetic process, cellular response to osmotic stress, acetate metabolic process, oxalate metabolic process, cellular response to oxidative stress, establishment or maintenance of cell polarity, UDP-glucose metabolic process, proteasomal ubiquitin-independent protein catabolic process, and galactose metabolic process (Fig. 1B and Supplementary Table S3).

To screen for the Vdps specific to sexual development, the protein spots showing more than 2-fold reduction in the Δ*veA* strain during sexual development were selected for functional analysis. Cellular functions of most Vdps, except CatB and FbpA, have not been previously characterized and their predicted roles, based on the GO annotation of AspGD (www.AspGD.org), are summarized in Table 1. To investigate the function of Vdps during development of *A. nidulans*, their corresponding genes (*svfA*, *vdpC*, *vdpF*, and *vdpJ*) were deleted and these deletions were confirmed by PCR and Southern blotting (Supplementary Fig. S2 for the *svfA*-deletion). When parameters like vegetative growth, responses to osmotic-, temperature-, oxidative-, cell wall- and cell membrane-stresses, and asexual and sexual development were examined, no significant abnormalities were seen in these deletion strains except *svfA*-deletion strain (Δ*svfA*) (Supplementary Fig. S3). Thereafter, Δ*svfA* strain was used for further investigations.

**Table 1:** VeA-dependent proteins (Vdps) selected from 2DE.

Name	Locus tag	SSP	Predicted function	V9^a^	S6^a^	tMW^b^	tPI^c^	MP^d^	Cov.^e^
*svfA*	*AN0117*	1611R	Oxidative stress protein, survival factor	3.13	−2.86	53.35	4.8	12/14	15
		1621R		0.00	2.90	51.93	4.8	14/14	18
*vdpB*	*AN1763*	5512 R	Oxidoreductase, short-chain dehydrogenase/reductase	2.32	−2.60	46.49	5.9	8/11	28
*vdpC*	*AN3331*	4428 R	Hypothetical protein	0.00	−2.46	41.40	5.4	9/10	33
*vdpD*	*AN5793*	2320 R	Proteasome subunit alpha type 3	0.00	−2.86	33.96	5.1	3/3	11
*vdpE*	*AN7267*	5712 R	Conserved hypothetical protein	0.00	−2.21	63.25	5.8	12/12	28
*vdpF*	*AN0567*	8809 R	Putative alcohol oxidase	1.32	−1.38	80.40	8.7	14/14	25
*vdpH*	*AN1152*	2115 R	Hypothethical protein	2.99	−0.31	13.52	5.1	4/4	40
		3123 R		0.00	−3.27	15.53	5.4	3/3	27
*vdpI*	*AN3168*	0407 R	Vacuolar ATP synthase subunit D	0.00	−2.79	41.95	4.3	7/7	19
*vdpJ*	*AN4532*	2323 R	Catechol oxygenase	0.00	−3.11	30.29	5.1	10/10	41
*fbpA*	*AN5604*	5511 R	Fructose-bisphosphatase	0.00	−3.28	45.19	5.9	8/16	21
*catB*	*AN9339*	0918 R	Catalase B precursor	0.00	−3.05	102.11	4.4	22/22	35
		0922 R		0.00	−2.19	99.01	4.4	15/15	21
		0923 R		2.60	−0.95	102.63	4.5	22/22	33
		0924 R		2.05	−1.23	98.92	4.5	24/24	42

^a^Levels of expression compared Δ*veA* to *veA*^+^ strains under each developmental stage (V9: vegetative stage 9 h, S6: sexual stage 6 h). ^**b**^Theoretical mass (kDa). ^**c**^Theoretical PI. ^**d**^Number of peptides of which mass matched theoretical numbers of peptides. ^**e**^Sequence coverage (%) in PMF.

### SvfA regulates vegetative growth and functions in oxidative- and cold-stress responses

**Figure 2:**
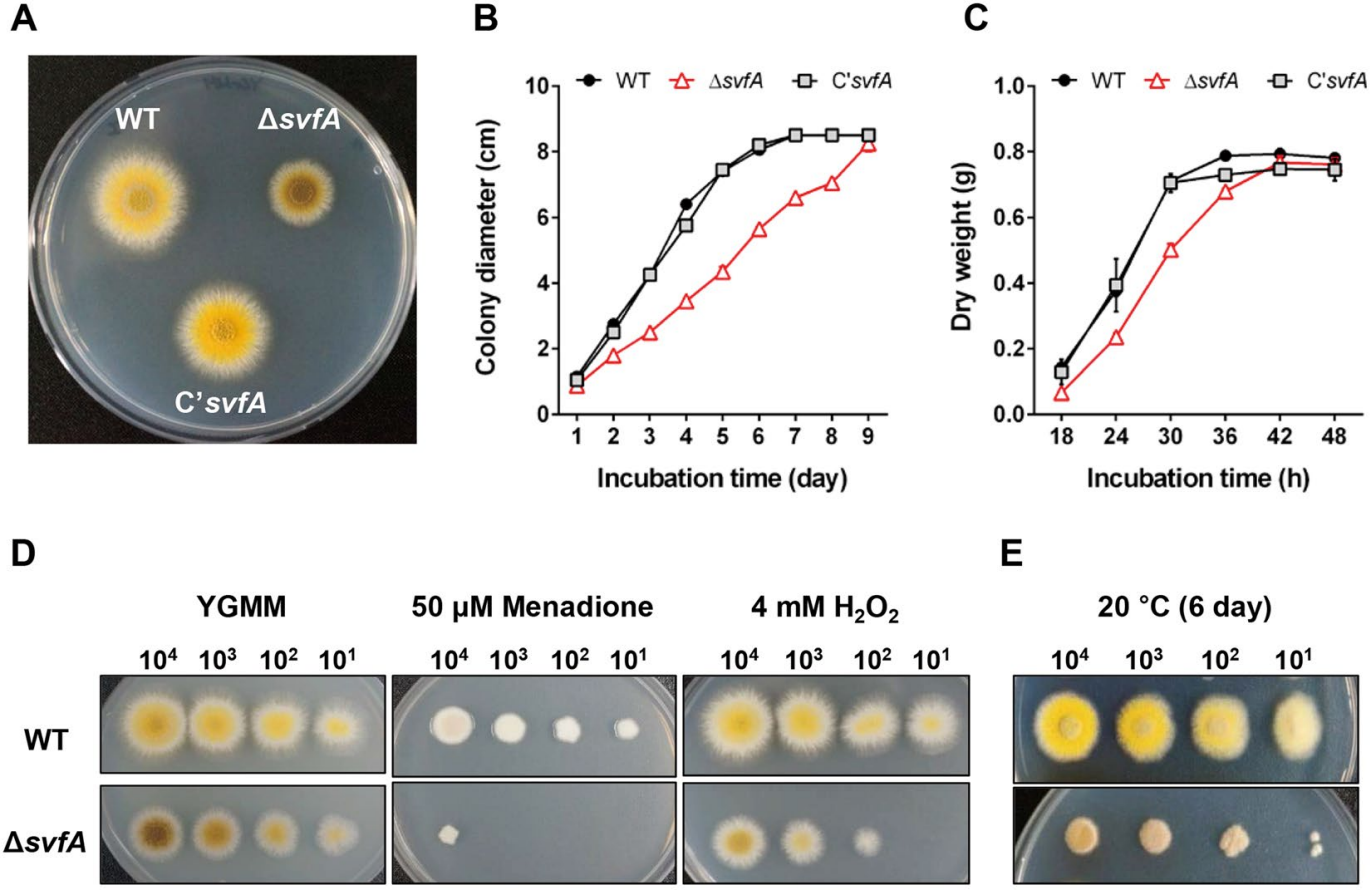
Growth patterns of different strains. (**A**) Colony morphology. Spores of WT, Δ*svfA*, and C’*svfA* strains were inoculated on YCMM plate and incubated for 2 days at 37 °C. (**B**) Radial growth. Over 9 days, the colony diameter of each culture from the point of inoculation was measured daily. (**C**) Mycelial production. Over 48 h, dry weight of each culture from the point of inoculation in liquid medium was measured. (**D**) Sensitivity to oxidative stress. Spores with 10-fold serial dilutions were spotted on YCMM containing menadione and hydrogen peroxide (H_2_O_2_) and incubated at 37 °C for 2 days. (**E**) Sensitivity to cold stress. Spores with 10-fold serial dilutions were spotted on YCMM at 20 °C for 6 days.

The Δ*svfA* mutant showed retarded radial growth on solid medium (Fig. 2A,B) and reduction in biomass production (Fig. 2C) with smaller mycelial balls (data not shown) in liquid culture. These growth defects were reversed by the re-introduction of the *svfA* gene (Fig. 2A–C for C’*svfA*; complementation strain). In *S. cerevisiae*, the Svf1 protein is required for survival under conditions of oxidative stress and cold stress^24^. When the sensitivity of the Δ*svfA* mutant to menadione, H_2_O_2_, and low temperature (20 °C) was tested, the Δ*svfA* mutant was sensitive to chemical induction of reactive oxygen species (ROS) (Fig. 2D) and cold stress (Fig. 2E). These data suggested that, as in *S. cerevisiae*, SvfA function was required for survival of *A. nidulans* during oxidative stress and cold stress.

### SvfA affects conidial germination

**Figure 3:**
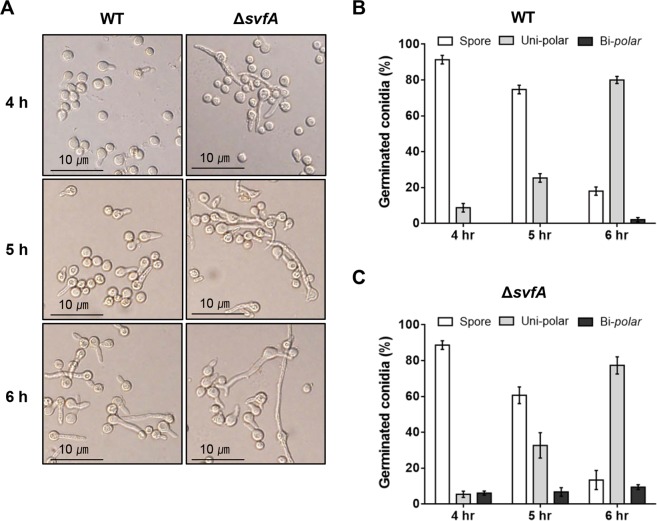
Roles of SvfA in conidial germination. (**A**) Morphology of germ tube. Conidia of the WT and Δ*svfA* strains were incubated in liquid GMM for the indicated time. The proportion of uni-polar and bi-polar germination of conidia in the WT (**B**) and Δ*svfA* strains (**C**). A total of 50 cells counted at each time point in triplicates, and results are shown as percentages.

When germination was observed in a time-dependent manner in GMM medium (minimal medium containing 1% glucose), WT conidia formed an unipolar germ tube after 4 h incubation (8.7% at 4 h, 25.3% at 5 h, and 80% at 6 h), and a few (2%) bipolar germ tubes in about 6 h (Fig. 3A,B). In contrast, Δ*svfA* conidia formed abnormally long, bipolar germ tubes from the beginning (6% at 4 h, 6.7% at 5 h, and 9.3% at 6 h) (Fig. 3A,C). When germination was observed in MM broth without glucose, unipolar germination was observed in both WT and Δ*svfA*, after 7 h incubation (Supplementary Fig S4). These results suggest that SvfA influences the establishment of polarity, but not the initiation of conidia germination.

### SvfA modulates conidiophore development and conidia production

**Figure 4:**
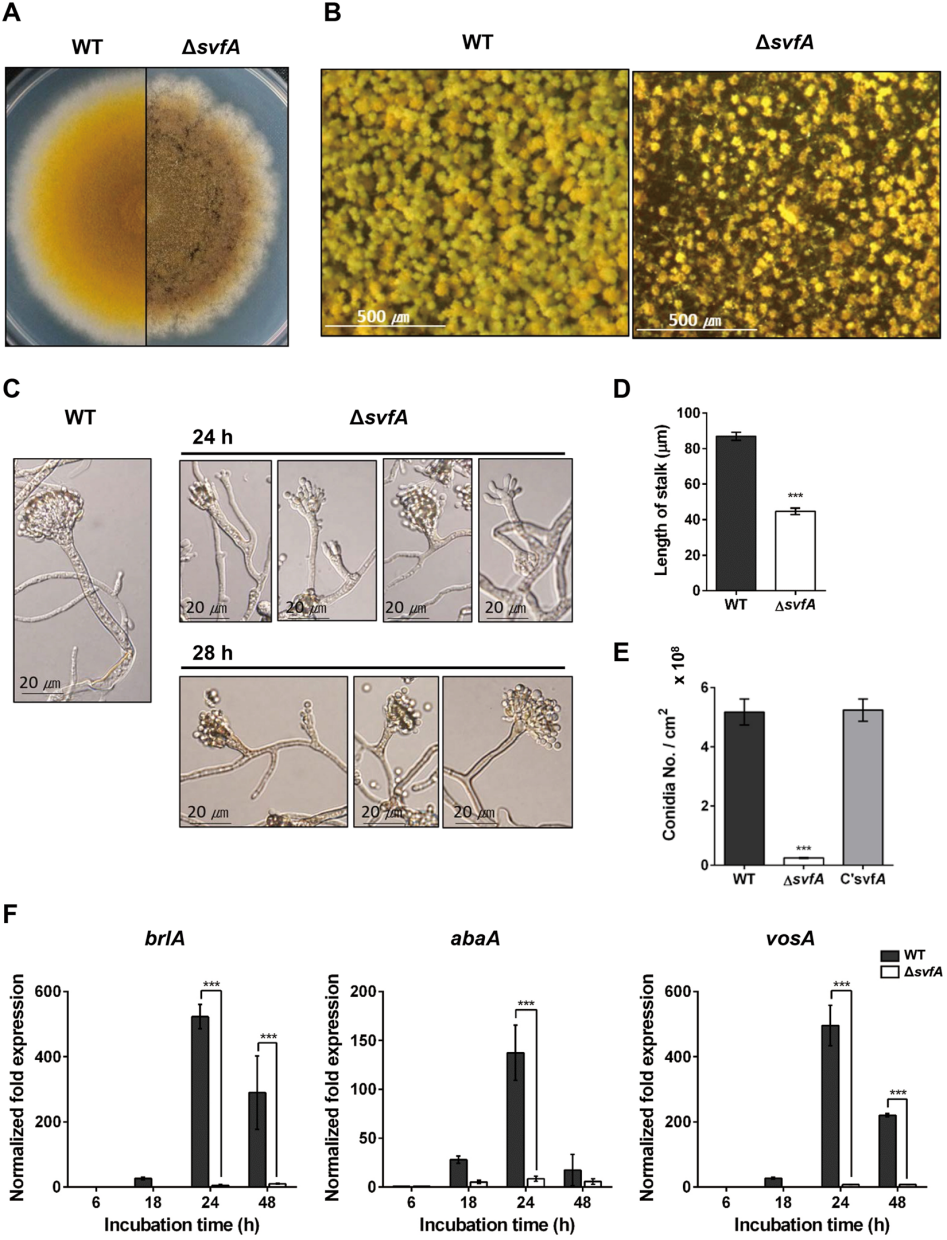
Effect of *svfA*-deletion on asexual development. (**A**) Colony morphology. Conidia of the WT and Δ*svfA* strains were inoculated on solid YCMM and incubated at 37 °C for 5 days (WT) and 7 days (Δ*svfA*) until the sizes of the colonies became similar. (**B**) Conidiophore heads on solid medium (**A**) as seen under a stereomicroscope. (**C**) Asexual reproductive organs. Conidia were inoculated on solid agar blocks, and the WT strain was incubated for 24 h whereas the Δ*svfA* strain was incubated for 24 h and 28 h, separately. (**D**) Length of stalks. Mean value of about 50 conidiophores. ^∗∗∗^P < 0.001. (**E**) Number of conidia produced. Average of triplicate readings is shown. ^∗∗∗^P < 0.001. (**F**) Expression patterns of genes *brlA, abaA*, and *vosA*, which are associated with asexual development. Mycelial balls of strains obtained from YCMM liquid culture were shifted to solid GMM to induce asexual development. Total RNA was extracted and RT-qPCR analysis was performed using 18 S rRNA gene as an internal control. ^∗∗∗^P < 0.001.

As indicated, the Δ*svfA* mutant grew slower than the WT strain, taking 7 days to reach the size of a 5-day WT colony. In addition, colony margins were irregular and colony color changed from yellow to faded-brownish yellow in the Δ*svfA* strain (Fig. 4A). Under the stereomicroscope, conidiophore heads in the Δ*svfA* strain showed reduction in number and size compared to the WT (Fig. 4B). Microscopic observation revealed a wide variety of abnormalities in conidiophore formation due to lack of SvfA, such as short stalks, branched stalks with abnormal head, and unstructured sterigmata layers (Fig. 4C); all these led to a reduction in conidiospore production. Statistical analysis revealed an approximately 50% decrease in stalk length (Fig. 4D) and 95% reduction in conidiospore production in the Δ*svfA* strain (Fig. 4E). In *A. nidulans*, a central regulatory pathway that controls asexual development is composed of three transcription factors, BrlA, AbaA, and WetA^25^. The transcript levels of these genes, along with that of *vosA*, which codes for one of the velvet family proteins involved in spore viability, were evaluated by RT-qPCR. Mycelial balls, produced in liquid YCMM, were shifted to solid MM to induce synchronized asexual development, and RNA was isolated from the cultures at the indicated time, post-induction. The WT strain expressed *brlA* and *vosA*, with peaks at 24 and 48 h, and *abaA* with a peak at 24 h, while the Δ*svfA* strain showed significant reduction in the transcript levels of *brlA*, *abaA*, and *vosA* (Fig. 4F). As VosA is reported to affect conidiospore viability^9^, conidia were collected from WT, Δ*svfA*, and C’*svfA*, grown for 2, 5, and 7 days, and their viability was tested. However, no significant difference among strains was observed (Supplementary Fig. S5). Taken together, these results indicate that SvfA regulates asexual development by regulating the induction of genes that are critical for this process.

### SvfA is essential for the completion of sexual development

**Figure 5:**
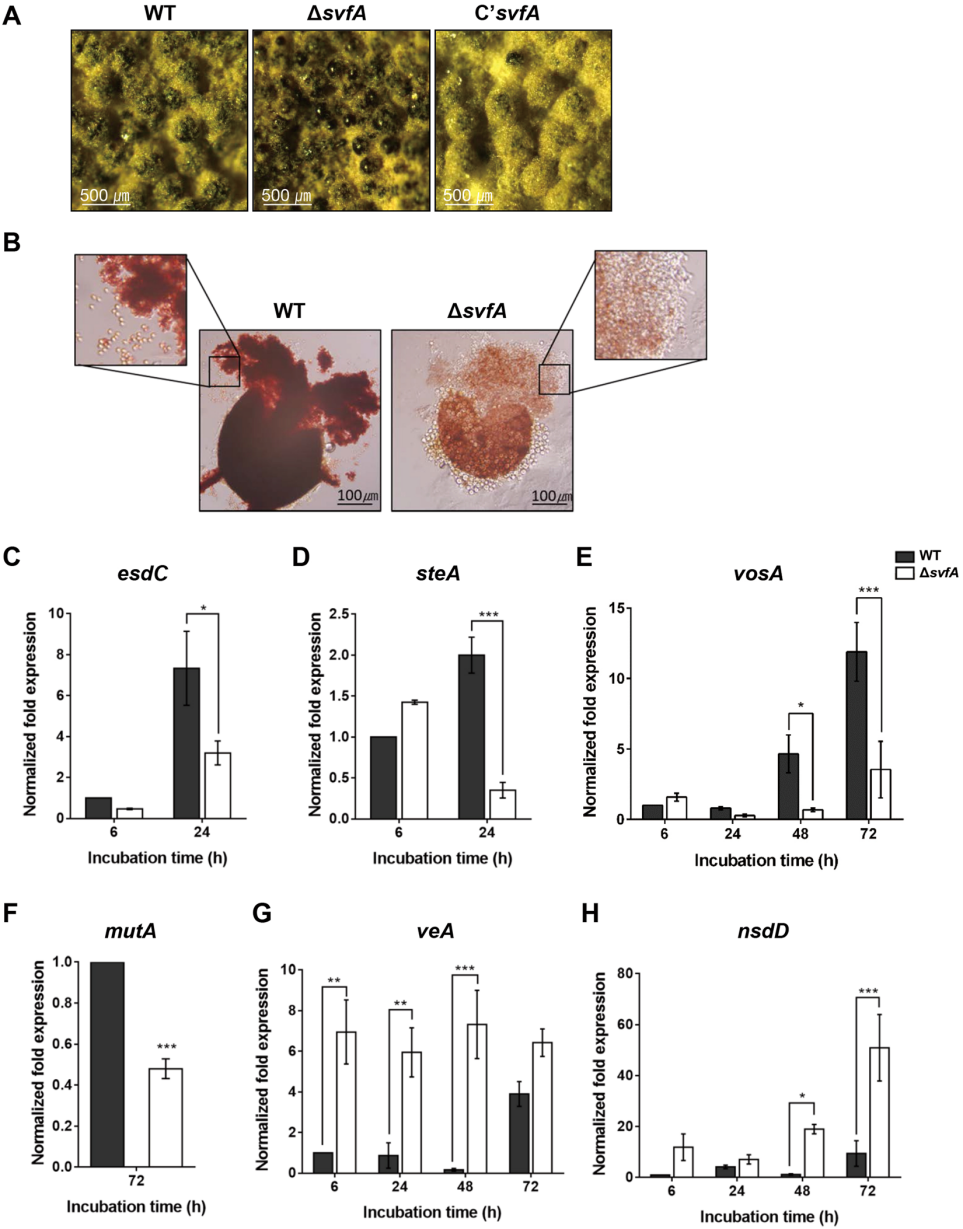
Effect of *svfA*-deletion on sexual development. (**A**) Sexual reproductive organs produced on MMCA solid medium. Mycelial balls of WT, Δ*svfA*, and C’*svfA* strains were shifted to solid MMCA medium and incubated for 3 days under conditions which induce sexual development. The images were captured under a stereomicroscope. (**B**) Cleistothecia and ascospores. A cleistothecium was ruptured on a glass slide to observe ascospores (enlarged image). (**C–H**) RT-qPCR analyses of genes during sexual development using 18 S rRNA gene as internal control. Expression patterns of *esdC* (**C**) and *steA* (**D**), which are genes involved in early sexual development. Expression patterns of the *vosA* gene for spore viability (**E**) and the *mutA* gene for the mutanase expressed mainly in Hülle cells (**F**). Expression patterns of *veA* (**G**) and *nsdD* (**H**), which are developmental regulators that activate sexual development. ^∗^P < 0.05; ^∗∗∗^P < 0.001.

To investigate the effect of *svfA-*deletion on sexual differentiation, strains were induced to undergo sexual development under dark and hypoxic conditions. The Δ*svfA* mutant produced small cleistothecia surrounded by few Hülle cells; this phenotype was rescued by the restoration of a functional *svfA* gene (Fig. 5A). When cleistothecia were ruptured to observe the formation of asci and ascospores, no ascospore production was observed in the Δ*svfA* strain (Fig. 5B). Unlike asexual development, which is controlled by a relatively simple central regulatory pathway^25^, the mechanism that regulates sexual development is much more complicated in *A. nidulans*^26^. For example, *esdC* and *steA* play roles in the early sexual stage, and *vosA* and *mutA* in the late sexual stage. EsdC has a glycogen binding domain, which is conserved in the beta subunit of the AMPK complex, and plays critical roles in promoting sexual development and regulating conidiation^27^. SteA is a transcription factor required for sexual development^28^, while VosA is required for the integrity of both asexual and sexual spores. This probably is the cause of defective cleistothecia, containing very few (~1%) viable ascospores in the Δ*vosA* mutant^9^. Alpha-1,3 glucanase MutA (mutanase) is expressed in the Hülle cells to nourish cleistothecia^29^. When the transcription of early sexual genes, *esdC* and *steA*, were analyzed at two different post-induction time-points, transcript levels of both genes were found to be decreased at 24 h in the Δ*svfA* strain (Fig. 5C,D). Transcription of late sexual gene *vosA* reduced by 7-fold at 48 h and 3-fold at 72 h (Fig. 5E), and that of the *mutA*, another late gene, decreased at 72 h (Fig. 5F). These results indicate that SvfA is a novel regulator that is essential for the completion of sexual development in *A. nidulans*, and is involved in the transcription of genes associated with early and late sexual development. To examine the possibility of sexual defects caused by insufficient arginine, we studied the phenotypes of the Δ*svfA* strain in medium supplemented with arginine. Addition of arginine did not alleviate the defects in colony morphology, asexual development, or sexual development (Supplementary Fig. S6).

### SvfA is linked to other developmental processes

Next, we examined the expression of genes for upstream sexual regulators, *veA* and *nsdD*. VeA, the founding member of the velvet family, is highly conserved in dimorphic and filamentous fungi^11^ and activates sexual development and secondary metabolism^4,18^. NsdD is a GATA type transcription factor required for sexual development, and the gene *nsdD* is highly expressed in the vegetative and asexual stages of *A. nidulans*, when compared to the sexual stages^30^. In the Δ*svfA* mutant, both *veA* and *nsdD* genes showed very high expression, unlike in the WT (Fig. 5G,H), suggesting that SvfA is required for down-regulation of *veA* and *nsdD* to regulate sexual development temporally. Development in *A. nidulans* is also closely connected to secondary metabolism^16^. To confirm this, the effect of *svfA-*deletion on sterigmatocystin (ST) production was tested by thin-layer chromatography (TLC) analysis. The Δ*svfA* strain produced a lower amount of ST compared to WT as well as C’*svfA* strains grown on solid GMM for 4 days (Supplementary Fig. S7A). To monitor the time-course profile of ST production, mycelial balls from liquid GMM were transferred to solid GMM and incubated for 6, 18, 24, and 48 h. The Δ*svfA* mutant showed a significantly lower amount of ST production during asexual development, compared to the WT strain (Supplementary Fig. S7B,C). These results suggested that SvfA is required for ST production.

### Cytoplasmic localization of SvfA is light-independent

**Figure 6:**
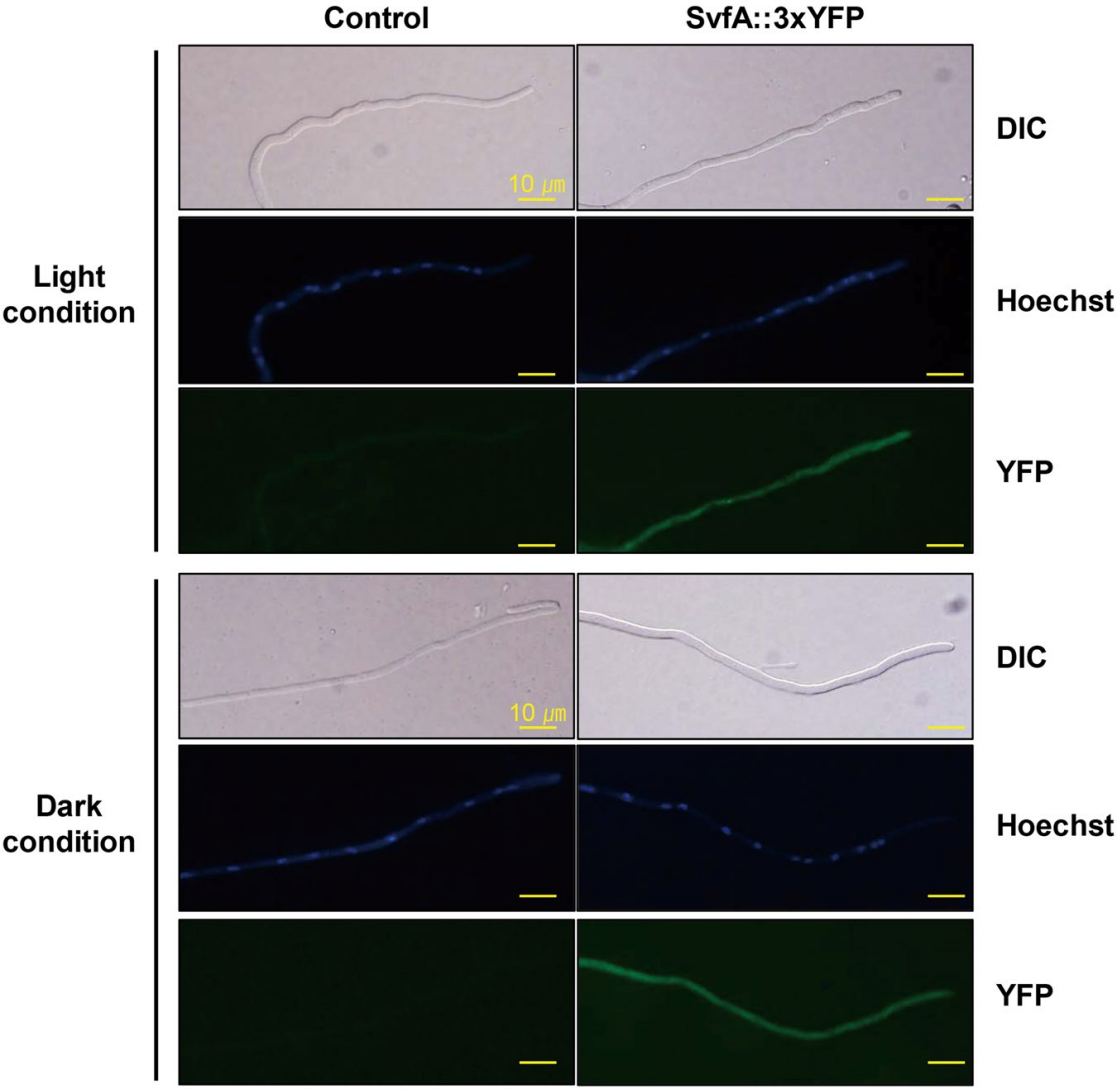
Intracellular localization of SvfA. Localization of SvfA was conducted by expressing SvfA::3xYFP fusion protein. Germlings were grown on coverslips submerged in liquid GMM overnight. Under both light and dark conditions, SvfA was localized to the cytoplasm.

To localize SvfA in the cell, an AYA strain expressing the SvfA fused to a 3xYFP C-terminal tag in the Δ*svfA* background was constructed. The AYA strain complemented the *svfA*-deletion phenotype (Supplementary Fig. S8A). The *svfA* expression pattern of the AYA strain was similar to that of the WT (Supplementary Fig. S8B). Strains were cultured on coverslips on solid MM at 30 °C for 24 h under light and dark conditions separately. Under both these conditions, SvfA-YFP fusion protein was localized to the cytoplasm and homogenously distributed in the hyphae (Fig. 6).

### VeA affects production of a larger form of SvfA protein during sexual development

**Figure 7:**
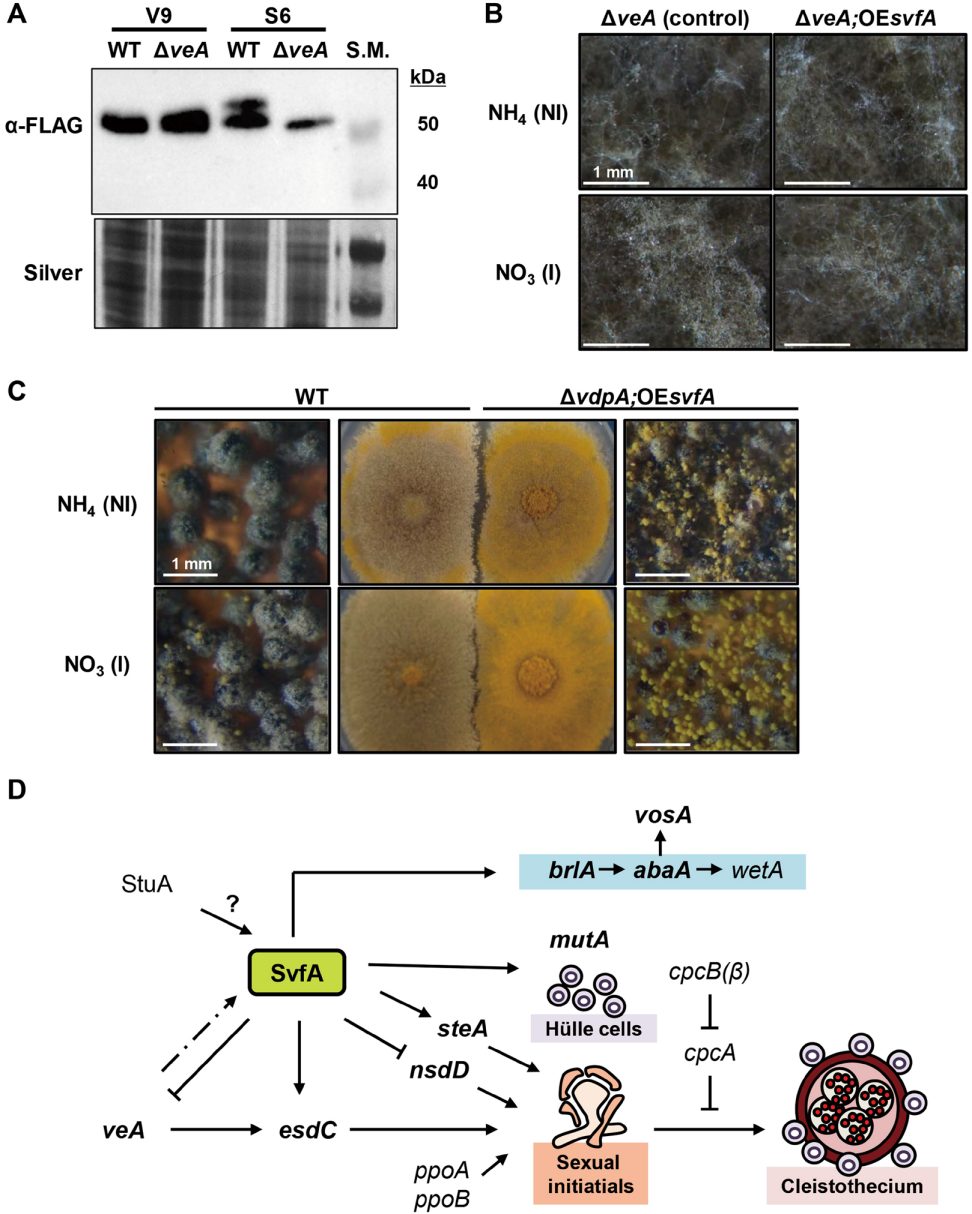
Effect of SvfA overexpression on sexual development and expression pattern of *svfA* during sexual development. (**A**) Upper panel: photograph of Western blotting of WT and Δ*veA* strains. The SvfA::FLAG fusion proteins were detected using an anti-FLAG antibody at the predicted size of approximately 54.3 kDa and 52.9 kDa. The capital letters V and S indicate the vegetative stage (V) and sexual development (S). Numbers indicate incubation time (h). Lower panel: photograph of SDS-polyacrylamide (8%) gel of total proteins visualized by silver staining. (**B**) Micrographs of cultured mycelia. Mycelial balls were transferred onto non-inducing medium (GMM containing 0.2% ammonium tartrate as a nitrogen source) or inducing medium (GMM containing 0.6% sodium nitrate as a nitrogen source) and incubated at 37 °C for 6 days under conditions which induce sexual development. Images were captured under a stereomicroscope. (**C**) Colonies of the WT and Δ*svfA;*OE*svfA* strains grown for 4 days with sealing and 2 additional days without sealing on non-inducing medium (GMM containing 0.2% ammonium tartrate as a nitrogen source) or inducing medium (GMM containing 0.6% sodium nitrate as a nitrogen source). (**D**) Proposed model for the involvement of SvfA in asexual and sexual development in *A. nidulans*. SvfA activates *brlA* gene expression and thus affects expression of downstream effectors *abaA* and *vosA* during asexual development. During sexual development, VeA-dependent post-translational modification activates SvfA, which in turn down-regulates the expression of *veA* and *nsdD*, and up-regulates the expression of *esdC, steA*, *vosA*, and *mutA*. SvfA is possibly regulated through the StuA for temporal regulation of both asexual and sexual development. Arrowheads denote positive regulation and flat arrows denote negative regulation. Solid lines denote transcriptional regulation and dash-dotted line denotes translational regulation. Genes, of which transcription were affected by SvfA, are emboldened.

To investigate the effect of VeA on SvfA protein production during sexual development of *A. nidulans*, recombinant strains expressing SvfA fused to a FLAG C-terminal tag in the WT and Δ*veA* backgrounds were generated. Western blot using anti-FLAG antibody revealed that the larger form was expressed only in the WT strain during sexual development (S6) and that the smaller form was expressed in the WT and Δ*veA* strains during both vegetative and sexual development (Fig. 7A), suggesting that VeA positively modulates production of the sexual-specific larger form of SvfA. In 2-DE analysis, two different protein spots, both identified as SvfA, showed the same isoelectric point (pI 4.8) but different molecular weights (MW 53.35 and 51.93 kDa) and an opposite pattern in intensity changes at the sexual stage (Table 1). These results suggest possible VeA-dependent post-translational modifications, such as glycosylation, which affects the MW but not the pI of the SvfA protein during early sexual development. To study the functional relationship between *svfA* and *veA*, *svfA* was overexpressed in the Δ*veA* strain to investigate whether the defects in sexual development caused by *veA*-deletion were reversed. Under the conditions that induced sexual development, the Δ*veA;*OE*svfA* strain did not alleviate the sexual defects, i.e., failed to produce cleistothecia (Fig. 7B). Furthermore, the gene expression pattern of *svfA* in the Δ*veA* strain showed no change at 6, 24, and 48 h, and 2-fold increase at 72 h, compared to that in the WT, as indicated by RT-qPCR (Supplementary Fig. S9). Next, the effect of *svfA* overexpression on sexual development was determined. Unlike the complementation strain (C’*svfA*), which showed normal sexual development (Fig. 5A), the *svfA*-overproducing strain (OE*svfA*) showed defects similar to those seen in the Δ*svfA* strain, which produced small cleistothecia (Fig. 7C). Although the Δ*svfA* strain failed to produce ascospores, cleistothecia of the OE*svfA* strain contained a few ascospores (Supplementary Fig. S10). Taken together, these data indicate that SvfA functions downstream to VeA together with other VeA-regulated proteins and that normal level of *svfA* gene expression is crucial for progression and completion of sexual development in *A. nidulans*.

## Discussion

In the present study, we identified SvfA, a yeast survival factor 1 (Svf1) homologous protein, as a candidate for novel VeA-dependent protein associated with sexual development in *A. nidulans*, and provided evidence of its multiple roles in growth and developmental processes.

The survival factor 1 protein was first identified in *S. cerevisiae* and is required for survival under conditions of oxidative stress and cold stress^24^. Expression of the *svf1* gene in mammalian cells protects them from oxidative stress, which suggests that Svf1 can play a role in protecting yeast cells from ROS, preventing possible cell death^24^. A recent report also revealed that survival factor *Ss*Svf1 is required for oxidative stress response and full virulence in plant pathogenic fungus *Sclerotinia sclerotiorum*; *SsSvf1-*gene silenced strains showed overproduction of ROS, impaired cell wall integrity, and reduced virulence^31^. The *A. nidulans svfA-*deletion (Δ*svfA*) strain showed sensitivity to chemical induction of reactive oxygen species (Fig. 2D). Although the molecular mechanisms underlying survival factor functions in fungi remain unclear^24,31^, the results presented in our study showed that SvfA is required for survival under oxidative stress in *A. nidulans*, as in *S. cerevisiae* and *S. sclerotiorum*. It is also noteworthy that amino acid homology searches indicated the presence of Svf1 homologous sequences in plant pathogenic fungi^40^ and other fungi including human pathogenic fungi (Supplementary Fig. S11).

Normal conidiophore morphogenesis requires functional interactions between transcription factors such as BrlA, AbaA, and StuA^1,27^. The Δ*svfA* strain formed structurally abnormal conidiophores and thus produced lower amounts of conidiospores due to the reduction in the transcript levels of *brlA*, *abaA*, and *vosA* (Fig. 4F), indicating that SvfA regulates the induction of genes for transcription factors that are critical for asexual development. A rather complicated mechanism is involved in regulation of sexual development^26^, with *esdC* and *steA* genes being expressed at early sexual stage, while *vosA* and *mutA* are expressed at later sexual stages. The *svfA-*deletion strain produced few Hülle cells and small cleistothecia with no ascospores due to the decreased transcription of *esdC*, *steA*, *vosA*, and *mutA*, indicating that SvfA is a novel regulator, essential for the completion of sexual development in *A. nidulans*. When we analyzed the expression of genes for upstream sexual regulators VeA and NsdD, expression of both *veA* and *nsdD* genes was highly increased by the *svfA-*deletion (Fig. 5G,H), suggesting that SvfA is required for negative feedback-regulation of *veA* and *nsdD* transcription to down-regulate sexual development temporally.

In consistency with the 2-DE-identification of Vdps, which revealed two protein spots of SvfA (Table 1), production of two forms of SvfA protein was revealed by Western blot analysis; production of the larger form is VeA-dependent and sexual-specific, but that of smaller one is VeA-independent and sexual-non-specific (Fig. 7A). Therefore, the SvfA-YFP fusion protein localized in the cytoplasm (Fig. 6) may be the smaller protein. In addition, overexpression of *svfA* in the Δ*veA* strain did not alleviate the sexual defects (i.e. failed to produce cleistothecia) (Fig. 7B) and overexpression of *svfA* in the WT showed defects similar to those seen in the Δ*svfA* strain, which produced small cleistothecia (Fig. 7C). These results indicate the following: post-translational modifications can occur to activate SvfA during sexual development, VeA is involved in this modification of SvfA, SvfA functions downstream to VeA together with other VeA-regulated proteins, and normal expression level of the *svfA* gene is crucial for progression and completion of sexual development in *A. nidulans*.

SvfA is involved in ST biosynthesis and development, both of which are known to be functionally interconnected^4^. CpcB, a Gβ-like protein, governs diverse cellular events such as germination, growth, development, and ST synthesis^32^. Similar to the Δ*svfA* strain, the *cpcB* deletion strain produced small and fragile cleistothecia with no ascospores and showed upregulation of *veA* and *nsdD* expression during sexual development^32^, suggesting a possible interaction between SvfA and CpcB. When levels of *cpcB* gene expression in the Δ*svfA* strain and *svfA* gene expression in the Δ*cpcB* strain were studied during sexual development, *cpcB* expression was not affected by the lack of SvfA and in turn *svfA* expression was not affected in Δ*cpcB* mutants (data not shown).

StuA, an APSES domain transcription factor, affects spatial organization of the conidiophores and is also required for the formation of Hülle cells and cleistothecia during early sexual development^1,33^. The *stuA* mutant has greatly shortened conidiophores lacking normal metulae and phialides^34,35^, which are quite similar to those from the Δ*svfA* strain. Interestingly, sequence analysis of the 5′-upstream region of *svfA* revealed 5′-(A/T)CGCG(T/A)N(A/C)-3′ for putative StuA Response Element (StRE)^36^ at positions −917, −744, −703, −701, −100, relative to the ATG codon. Although further experiments are required to reveal the relationship between StuA and SvfA, these results hint the possibility of SvfA being necessary for the temporal regulation of both asexual and sexual development through functional interaction with StuA (Fig. 7D).

Although mechanism for the multiple functions of the oxidative stress protein SvfA in the developmental processes of *A. nidulans* remains unclear, several data support that oxidative stress or an imbalanced intracellular redox environment affects development. The cellular oxidation state is one of the physiological changes during early sexual development. For example, NoxA, a NADPH oxidase in *A. nidulans*, is involved in the production of ROS and cleistothecial development at the early stage^37^. Transcription of *noxA* gene is suppressed by SakA MAP kinase^37^, and deletion of SakA shows an increased number of prematurely developed cleistothecia^38^. A *trxA* deletion strain fails to produce cleistothecia under standard conditions. However, low GSH levels leads to the development of cleistothecia, whereas high GSH levels results in the formation of asexual conidiophores^39^. Activation of the expression of *cpeA*, a catalase-peroxidase gene, by StuA is also required for the formation of Hülle cells and cleistothecia during early sexual development^1,33^. It is also noted that Svf1 of *S. cerevisiae* is involved in cell survival by affecting the sphingolipid metabolism^40^ and is a substrate of serine/threonine protein kinase CK2, which is essential for life in all eukaryotes by regulating the cell cycle, tumorigenesis, and apoptosis^41^. Interestingly, exogenous expression of the human anti-apoptotic gene *Bcl-x*_*L*_, which regulates apoptosis and the cell cycle^42^, could functionally complement the defect of Svf1 in *S. cerevisiae*^24^. Mammalian Bcl-x_L_ regulates the intrinsic pathway of apoptotic cell death activated in β-cells under prolonged oxidative and endoplasmic reticulum stress^43^.

Taken together, we can propose a model for the involvement of SvfA in asexual and sexual development in *A. nidulans* (Fig. 7D). Further studies should be performed to provide insight into the molecular mechanisms of SvfA-mediated developmental processes; however, our data presented in the present study indicate that the oxidative stress protein SvfA is a novel central regulator of growth, differentiation, and secondary metabolism in *A. nidulans*.

## Methods

### Proteome analysis

*A. nidulans* strains FGSC A4 (*veA*^+^) and KVE9 (Δ*veA*::*argB*) were used for proteome analysis. Fungal techniques for culture condition, observation, transformation, genetic analyses, and phenotypic analyses were performed according to the previous report^2^. For protein extraction, the *A. nidulans* cultures were harvested by filtration through a Miracloth and ground to powder using liquid nitrogen. The grinded samples were homogenized directly by motor-driven homogenizer (PowerGen125, Fisher Scientific) in sample lysis buffer with 7 M urea, 2 M thiourea containing 4% (w/v) CHAPS, 1% (w/v) DTT and 2% (v/v) Pharmalyte (pH 3.5–10, Amersham Biosciences), and 1 mM benzamidine^44^. Proteins were extracted for 1 h at room temperature with vortexing. After centrifugation at 15,000 × g for 1 h at 15 °C, the supernatant was used for further experiment.

Two-dimensional gel electrophoresis (2-DE) analysis was carried out essentially as described previously^44,45^. In brief, 200 μg of samples was loaded to rehydrated IPG strips with a nonlinear pH gradient from 4 to 10. Isoelectric focusing (IEF) was performed at 20 °C. The second dimensional SDS-PAGE (20 × 24 cm, 10–16%) was performed using Höefer DALT 2D system (Amersham Biosciences). 2D gels were silver stained as described by Oakley *et al*.^46^ but the fixing and sensitization step with glutaraldehyde was omitted. Images were analyzed by the PDQuest (version 7.0, BioRad) software^45^. Protein spots were selected for the significant expression variation deviated over two-fold in its expression level compared with the control or normal sample. Protein spots were enzymatically digested in-gel in a manner similar to that previously described by Shevchenko *et al*.^47^ using modified porcine trypsin (Promega) and analyzed using an Ettan MALDI-TOF mass spectrometer (Amersham Biosciences). The search program ProFound, developed by The Rockefeller University (https://129.85.19.192/profound_bin/ WebProFound.exe), was used for protein identification by peptide mass fingerprinting. Spectra were calibrated with trypsin auto-digestion ion peaks m/z (842.510, 2211.1046) as internal standards^48^.

### Generation of recombinant strains

The *A. nidulans* strains employed in the present study are listed in Supplementary Table S1. To construct the disruption cassette, amplified *argB* gene product was inserted between 5′-and 3′ flanking regions of each *svfA*, *vpdC*, *vpdF*, and *vdpJ* genes as a selective marker using double-joint PCR^49^. The information of primers for fusion products are listed in Supplementary Table S2. Disruption cassettes were amplified with each set of nested primers and introduced into the TJ1–1 strain. The correct recombination in genomic DNA was confirmed by PCR and Southern blotting. To complement Δ*svfA*, the *svfA* gene region, including its predicted promoter, was amplified and cloned into pHS13, which contains 3/4 of the *pyroA* gene, a FLAG tag, and the *trpC* terminator^11^. For construction of the SvfA-YFP strain, the *svfA* gene region, including its predicted promoter, was amplified and cloned into pHS-YFP^50^. To generate *svfA*-overexpressing strains, the *svfA* ORF was cloned into pHS11 containing the *niiA* promoter. The resulting plasmid was introduced into the recipient strain.

### Media and culture conditions

Strains were maintained in *Aspergillus* minimal medium with glucose (GMM)^32^. The minimal medium supplemented with 0.15% yeast extract and 0.15% casamino acid (YCMM) was used as a complete medium for vegetative mycelial ball production. To observe germination of conidia, conidia were inoculated in liquid GMM and MM (without glucose) and observed every 1 h after incubation at 37 °C using a microscope. For phenotypic analysis during development, vegetative mycelial balls incubated in liquid YCMM for 16–18 h were transferred to GMM solid or MMCA (MM with 0.15% casamino acid) solid media to induce asexual or sexual development, respectively. For sexual development, the media were sealed with parafilm for 24 h and incubated further without sealing under dark conditions. To control the expression by *niiA* promoter, 0.2% ammonium tartrate or 0.6% sodium nitrate (as a nitrogen source) was added as non-inducing medium or inducing medium, respectively.

### Sensitivity test to oxidative and cold stresses

Spotting susceptibility assays was performed as described previously^51,52^ but modified to some extent. Conidia were resuspended to 2 × 10^6^ cells per ml in distilled water and prepared at 10-fold dilutions. For each dilution, 5 µl was spotted onto a YCMM agar plate (control) or plates containing 50 μM menadione and 4 mM H_2_O_2_ for oxidative stress, and the plates were incubated for 2 days at 37 °C. To test the response to cold stress, conidia were spotted on YCMM and incubated for 6 days at 20 °C.

### RNA preparation, cDNA synthesis, and quantitative real-time PCR

Cells at each of the developmental stages were ground using liquid nitrogen with a pestle and mortar^52^. Total RNA was extracted using Trizol according to the manufacturer’s protocols (Invitrogen). cDNA was synthesized using 4 μg extracted RNA, hexamer primer, and M-MLV reverse transcriptase (Enzynomics) as described in the manufacturer’s instructions. RT-qPCR was performed using a Bio-Rad CFX96 Real-Time PCR System (Bio-Rad) and a TOPrealTM qPCR 2X PreMIX Kit (Enzynomics). Transcript levels of target genes were normalized against those of 18 S rRNA using 2^−ΔCt^ method described previously^53^. The information of primers for RT-qPCR are listed in Supplementary Table S2.

### Western blot analysis

Protein extraction and western blot analysis were performed as previously described^2^. The *A. nidulans* cultures were ground using liquid nitrogen, and cells were resuspended in protein extraction buffer (50 mM Tris-HCl, pH 8, 150 mM NaCl, 1 mM EDTA, and 1% NP-40) with 2 mM phenylmethylsulfonyl fluoride (PMSF), 10 mM sodium fluoride, and 1 mM sodium vanadate. The supernatant was obtained after centrifugation at 15,000 × g at 4 °C for 30 min. Total protein samples were electrophoresed on 8% SDS-PAGE and subsequently electroblotted onto Hybond-P polyvinylidene difluoride (PVDF) membranes (GE Healthcare). The membrane was blocked with 5% skimmed milk, and protein detection was carried out using anti-FLAG (Sigma-Aldrich) and goat anti-mouse IgG-HRP (Santa Cruz Biotechnology) secondary antibody following the manufacturers’ protocols (ELPISBIO). SDS-PAGE and silver staining kit (ELPISBIO) were used for silver staining.

### Microscopy

For microcopy, an Olympus System microscope Model BX51 (Olympus) equipped with UPlanSApo 60X and UPlanFL 100X objective lenses (Olympus) and stereomicroscope Model SMZ800 (Nikon) were used. Images were captured with a DP71 digital camera (Olympus) and processed using the DP manager imaging software (Olympus). For microscopic observation of the fungal hyphae, each strain was coverslip-cultured on a block of appropriate agar medium or incubated in liquid GMM medium. The coverslips were stained with 1 mg/ml Hoechst 33342 (Sigma-Aldrich) for labeling DNA^2^. DAPI (high brightness) filter cubes (excitation filter: center wavelength 377 nm, emission filter: center wavelength 447 nm, Olympus) and FITC filter cubes (excitation filter: center wavelength 483 nm, emission filter: center wavelength 535 nm, Olympus) were used to observe the fluorescence of Hoechst and YFP, respectively^50^.

## Supplementary information

Supplemental Figures and Tables.

## References

[1] Wu J, Miller BL (1997). *Aspergillus* asexual reproduction and sexual reproduction are differentially affected by transcriptional and translational mechanisms regulating stunted gene expression. Mol. Cell. Biol..

[2] Kang EH, Kim J, Oh HW, Park HM (2013). LAMMER kinase LkhA plays multiple roles in the vegetative growth and asexual and sexual development of *Aspergillus nidulans*. PLoS One.

[3] Bok JW, Keller NP (2004). LaeA, a regulator of secondary metabolism in *Aspergillus* spp. Eukaryot. Cell.

[4] Bayram Ö, Braus GH (2012). Coordination of secondary metabolism and development in fungi: The velvet family of regulatory proteins. FEMS Microbiol. Rev..

[5] Adams TH, Wieser JK, Yu JH (1998). Asexual sporulation in *Aspergillus nidulans*. Microbiol. Mol. Biol. Rev.

[6] Han K (2003). Environmental factors affecting development of *Aspergillus nidulans*. J. Microbiol..

[7] Han KH (2009). Molecular genetics of *Emericella nidulans* sexual development. Mycobiology.

[8] Bayram Ö (2012). The *Aspergillus nidulans* MAPK module AnSte11-Ste50-Ste7-Fus3 controls development and secondary metabolism. PLoS Genet..

[9] Ni M, Yu JH (2007). A novel regulator couples sporogenesis and trehalose biogenesis in *Aspergillus nidulans*. PLoS One.

[10] Dhingra S, Andes D, Calvoa AM (2012). VeA regulates conidiation, gliotoxin production, and protease activity in the opportunistic human pathogen *Aspergillus fumigatus*. Eukaryot. Cell.

[11] Park HS, Ni M, Jeong KC, Kim YH, Yu JH (2012). The role, interaction and regulation of the velvet regulator VelB in *Aspergillus nidulans*. PLoS One.

[12] Park HS, Nam TY, Han KH, Kim SC, Yu JH (2014). VelC positively controls sexual development in *Aspergillus nidulans*. PLoS One.

[13] Stinnett SM, Espeso EA, Cobeño L, Araújo-Bazán L, Calvo AM (2007). *Aspergillus nidulans* VeA subcellular localization is dependent on the importin α carrier and on light. Mol. Microbiol..

[14] Bayram Ö (2008). VelB/VeA/LaeA complex coordinates light signal with fungal development and secondary metabolism. Science..

[15] Sarikaya Bayram Ö (2010). LaeA control of velvet family regulatory proteins for light-dependent development and fungal cell-type specificity. PLoS Genet..

[16] Palmer JM (2013). Secondary metabolism and development is mediated by LlmF control of VeA subcellular localization in *Aspergillus nidulans*. PLoS Genet..

[17] Han KH, Park JS, Chae KS, Han DM (2010). Simple identification of *veA1* mutation in *Aspergillus nidulans*. J. Microbiol..

[18] Kim HS (2002). The *veA* gene activates sexual development in *Aspergillus nidulans*. Fungal Genet. Biol..

[19] Kato N, Brooks W, Calvo AM (2003). The expression of sterigmatocystin and penicillin genes in *Aspergillus nidulans* is controlled by *veA*, a gene required for sexual development. Eukaryot. Cell.

[20] Sarikaya-Bayram Ö (2014). Membrane-bound methyltransferase complex VapA-VipC-VapB guides epigenetic control of fungal development. Dev. Cell.

[21] Kim, H. R., Chae, K. S., Han, K. H. & Han, D. M. The *nsdC* gene encoding a putative C_2_H_2_ -type transcription factor is a key activator of sexual development in *Aspergillus nidulans*. *Genetics* **182**, 771–783, 10.1534/genetics.109.101667 (2009). 10.1534/genetics.109.101667 PMC2710158 19416940

[22] Kim Y, Nandakumar MP, Marten MR (2007). Proteome map of *Aspergillus nidulans* during osmoadaptation. Fungal Genet. Biol..

[23] Wartenberg D (2012). Proteome analysis of the farnesol-induced stress response in *Aspergillus nidulans*-The role of a putative dehydrin. J. Proteomics.

[24] Brace JL, VanderWeele DJ, Rudin CM (2005). Svf1 inhibits reactive oxygen species generation and promotes survival under conditions of oxidative stress in *Saccharomyces cerevisiae*. Yeast.

[25] Boylan MT, Mirabito PM, Willett CE, Zimmerman CR, Timberlake WE (1987). Isolation and physical characterization of three essential conidiation genes from *Aspergillus nidulans*. Mol. Cell. Biol..

[26] Dyer PS, O’Gorman CM (2012). Sexual development and cryptic sexuality in fungi: insights from *Aspergillus* species. FEMS Microbiol. Rev..

[27] Han KH (2008). The *Aspergillus nidulans esdC* (early sexual development) gene is necessary for sexual development and is controlled by *veA* and a heterotrimeric G protein. Fungal Genet. Biol..

[28] Vallim, M. A., Miller, K. Y. & Miller, B. L. *Aspergillus* SteA (sterile12-like) is a homeodomain-C_2_/H_2_-Zn^+2^ finger transcription factor required for sexual reproduction. *Mol. Microbiol.* **36**, 290–301, 10.1046/j.1365-2958.2000.01874.x (2000). 10.1046/j.1365-2958.2000.01874.x 10792717

[29] Wei H, Scherer M, Singh A, Liese R, Fischer R (2001). *Aspergillus nidulans* α-1,3 glucanase (mutanase), *mutA*, is expressed during sexual development and mobilizes mutan. Fungal Genet. Biol..

[30] Han KH (2001). The *nsdD* gene encodes a putative GATA-type transcription factor necessary for sexual development of *Aspergillus nidulans*. Mol. Microbiol..

[31] Yu Y (2019). Survival factor 1 contributes to the oxidative stress response and is required for full virulence of *Sclerotinia sclerotiorum*. Mol. Plant Pathol..

[32] Kong Q (2013). Gβ-Like CpcB plays a crucial role for growth and development of *Aspergillus nidulans* and *Aspergillus fumigatus*. PLoS One.

[33] Scherer M, Wei H, Liese R, Fischer R (2002). *Aspergillus nidulans* catalase-peroxidase gene (*cpeA*) is transcriptionally induced during sexual development through the transcription factor StuA. Eukaryot. Cell.

[34] Miller KY, Wu JG, Miller BL (1992). StuA is required for cell pattern-formation in *Aspergillus*. Genes Dev.

[35] Miller KY, Toennis TM, Adams TH, Miller BL (1991). Isolation and transcriptional characterization of a morphological modifier: the *Aspergillus nidulans* stunted (*stuA*) gene. Mol. Gen. Gene..

[36] Park BC (2014). Transcriptional regulation of *fksA*, a β-1,3-glucan synthase gene, by the APSES protein StuA during *Aspergillus nidulans* development. J. Microbiol..

[37] Lara-Ortíz T, Riveros-Rosas H, Aguirre J (2003). Reactive oxygen species generated by microbial NADPH oxidase NoxA regulate sexual development in *Aspergillus nidulans*. Mol. Microbiol.

[38] Kawasaki L, Sánchez O, Shiozaki K, Aguirre J (2002). SakA MAP kinase is involved in stress signal transduction, sexual development and spore viability in *Aspergillus nidulans*. Mol. Microbiol..

[39] Sato I, Shimizu M, Hoshino T, Takaya N (2009). The glutathione system of *Aspergillus nidulans* involves a fungus-specific glutathione S-transferase. J. Biol. Chem..

[40] Brace JL, Lester RL, Dickson RC, Rudin CM (2007). *SVF1* regulates cell survival by affecting sphingolipid metabolism in *Saccharomyces cerevisiae*. Genetics.

[41] Masłyk M (2008). Yeast surviving factor Svf1 as a new interacting partner, regulator and *in vitro* substrate of protein kinase CK2. Mol. Cell. Biochem..

[42] Janumyan YM (2003). Bcl-x /Bcl-2 coordinately regulates apoptosis, cell cycle arrest and cell cycle entry. EMBO J..

[43] Aharoni-Simon M (2016). Bcl-2 regulates reactive oxygen species signaling and a redox-sensitive mitochondrial proton leak in mouse pancreatic β-cells. Endocrinology.

[44] Uhm YK (2010). Effects of *Machilus thunbergii* Sieb et Zucc on UV-induced photoaging in hairless mice. Phyther. Res.

[45] Deng WW, Sasamoto H, Ashihara H (2015). Effect of caffeine on the expression pattern of water-soluble proteins in rice (*Oryza sativa*) seedlings. Nat. Prod. Commun.

[46] Oakley BR, Kirsch DR, Morris NR (1980). A simplified ultrasensitive silver stain for detecting proteins in polyacrylamide gels. Anal. Biochem..

[47] Shevchenko A, Wilm M, Vorm O, Mann M (1996). Mass spectrometric sequencing of proteins silver-stained polyacrylamide gels. Anal. Chem..

[48] Lee SY, Lee KO (2011). Proteomic analysis of RNA interference induced knockdown plant. Methods Mol. Biol.

[49] Yu JH (2004). Double-joint PCR: a PCR-based molecular tool for gene manipulations in filamentous fungi. Fungal Genet. Biol..

[50] Kim YJ, Yeong Man Y, Maeng PJ (2017). Differential control of asexual development and sterigmatocystin biosynthesis by a novel regulator in *Aspergillus nidulans*. Sci. Rep.

[51] Rocha MC (2016). *Aspergillus fumigatus* MADS-Box transcription factor *rlmA* is required for regulation of the cell wall integrity and virulence. G3 (Bethesda).

[52] Rocha MC (2015). The *Aspergillus fumigatus pkcA* mutant is defective in the activation of the cell wall integrity pathway but is dispensable for virulence in a neutropenic mouse infection model. PLoS One.

[53] Park, D. S., Yu, Y. M., Kim, Y. J. & Maeng, P. J. Negative regulation of the vacuole-mediated resistance to K^+^ stress by a novel C_2_H_2_ zinc finger transcription factor encoded by *aslA* in *Aspergillus nidulans*. *J. Microbiol.* **53**, 100–110, 10.1007/s12275-015-4701-8 (2015). 10.1007/s12275-015-4701-8 25626364

[54] Benjamini Y, Hochberg Y (1995). Controlling the false discovery rate: a practical and powerful approach to multiple testing. J. R. Stat. Soc. Ser. B.

[55] Priebe S, Kreisel C, Horn F, Guthke R, Linde J (2015). FungiFun2: a comprehensive online resource for systematic analysis of gene lists from fungal species. Bioinformatics.

