# Supplementary material for: Survival factor SvfA plays multiple roles in differentiation and is essential for completion of sexual development in Aspergillus nidulans
Source: Sci Rep. 2020 Mar 27;10:5586. doi: 10.1038/s41598-020-62455-4 (PMC7101369; doi:10.1038/s41598-020-62455-4)
Supplement: Supplementary file 1 — Supplemental Figures and Tables. [file 41598_2020_62455_MOESM1_ESM.pdf]

Supplementary information

Survival factor SvfA plays multiple roles in differentiation and is essential for completion of sexual development in *Aspergillus nidulans*

Joo-Yeon Lim, Eun-Hye Kang, Yun-Hee Park, Jun-Ho Kook, and Hee-Moon Park*

Department of Microbiology and Molecular Biology,

College of Bioscience and Biotechnology,

Chungnam National University, Daejeon 34134, Korea

*** To whom correspondence should be addressed.**

Hee-Moon Park

Tel: +82-42-821-7553

Fax: +82-42-822-7367

E-mail: hmpark@cnu.ac.kr

Supplementary Figure S1

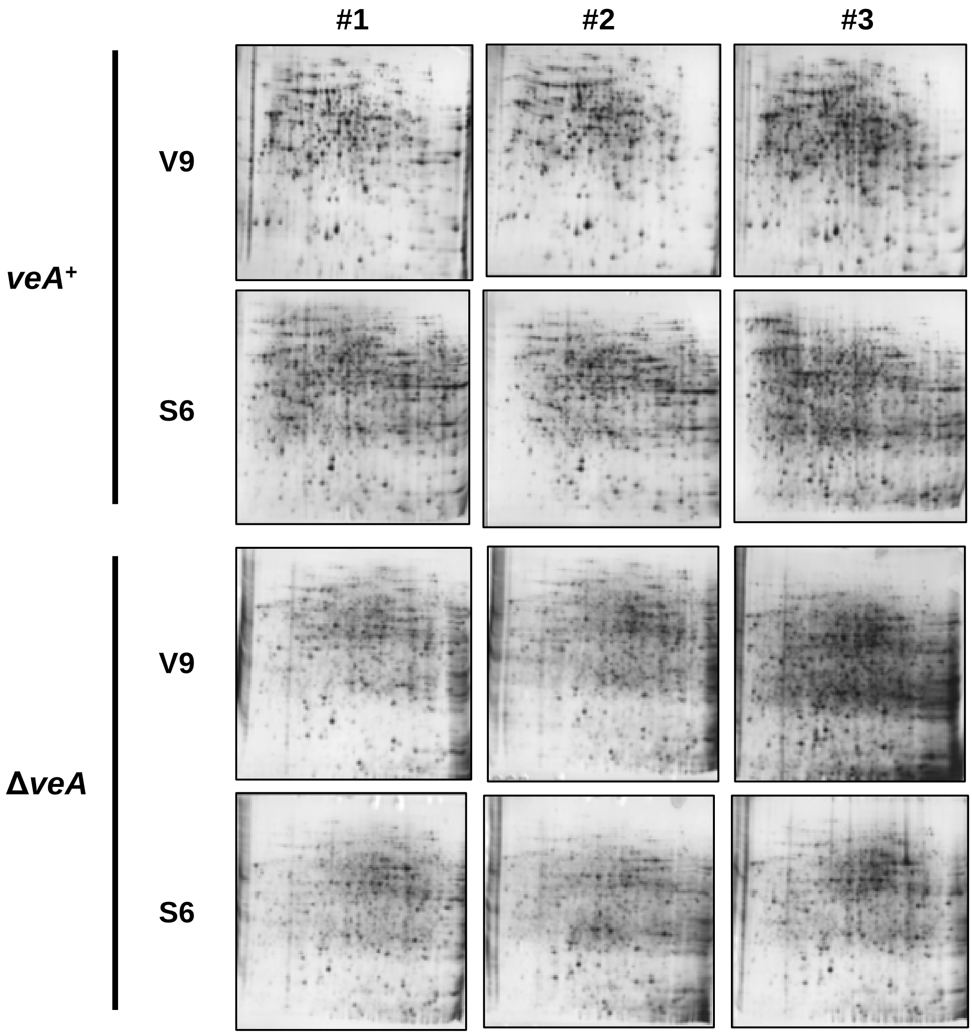

Supplementary Figure S1. Two-dimensional gel map. Equal amounts of total proteins of WT (*veA*$^{+}$) and Δ*veA* strains at 9 h incubation during vegetative growth (9 h; V9) and sexual development (6 h; S6) were separated. The 2-DE analyses were repeated three times using samples harvested independently.

Supplementary Figure S2

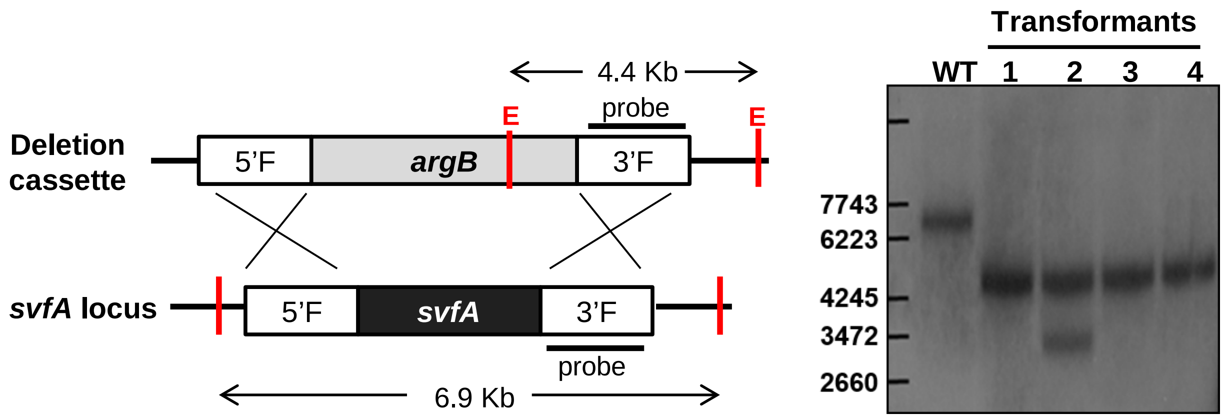

Supplementary Figure S2. Restriction map and Southern blot analysis.

Genomic DNA of WT and Δ*svfA* strains, digested with *Eco* RI, were separated on a 0.8% agarose gel, blotted, and hybridized with the probe. Transformant-1 was used for further analyses. 'E's denote the *Eco* RI sites.

Supplementary Figure S3

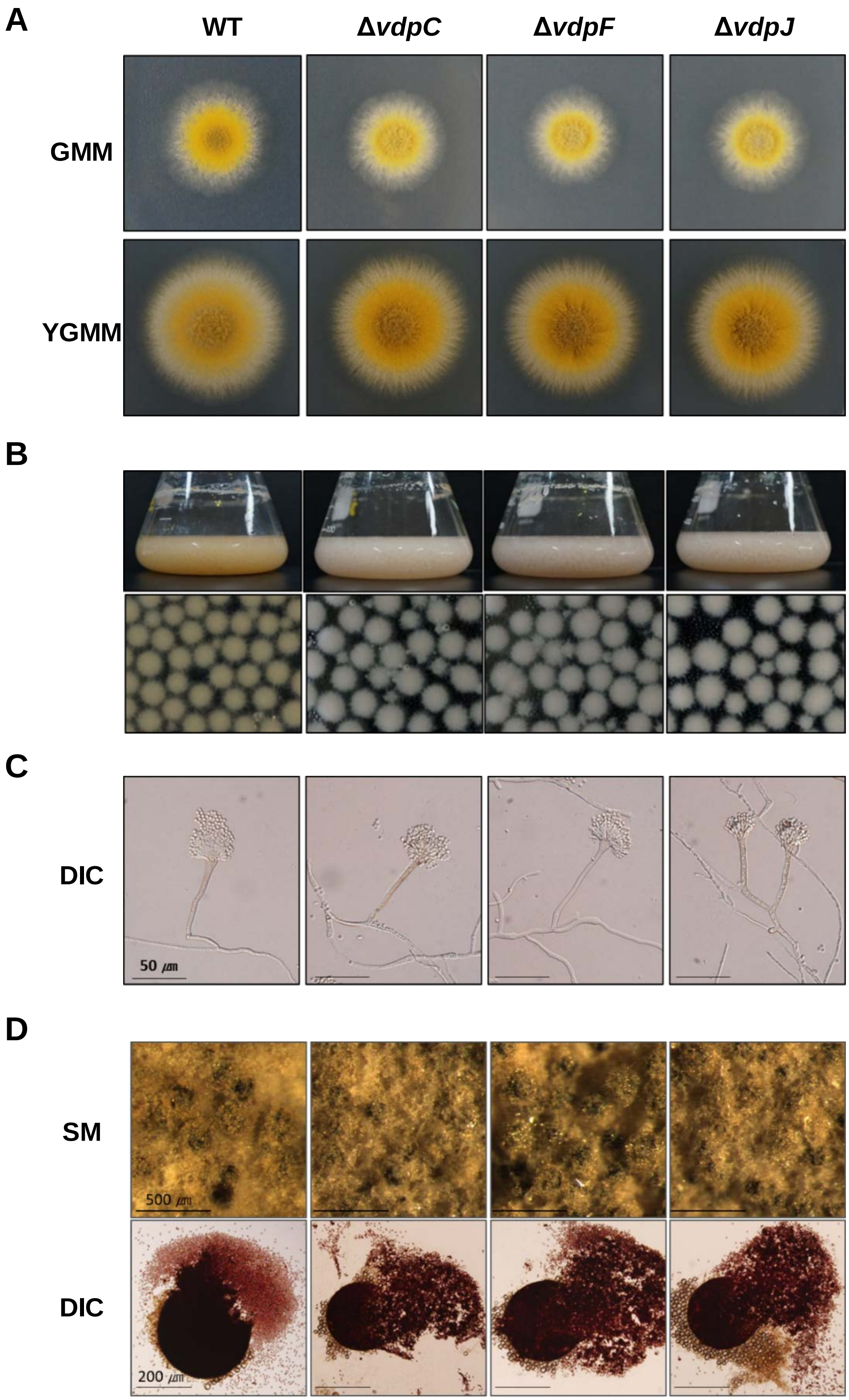

E

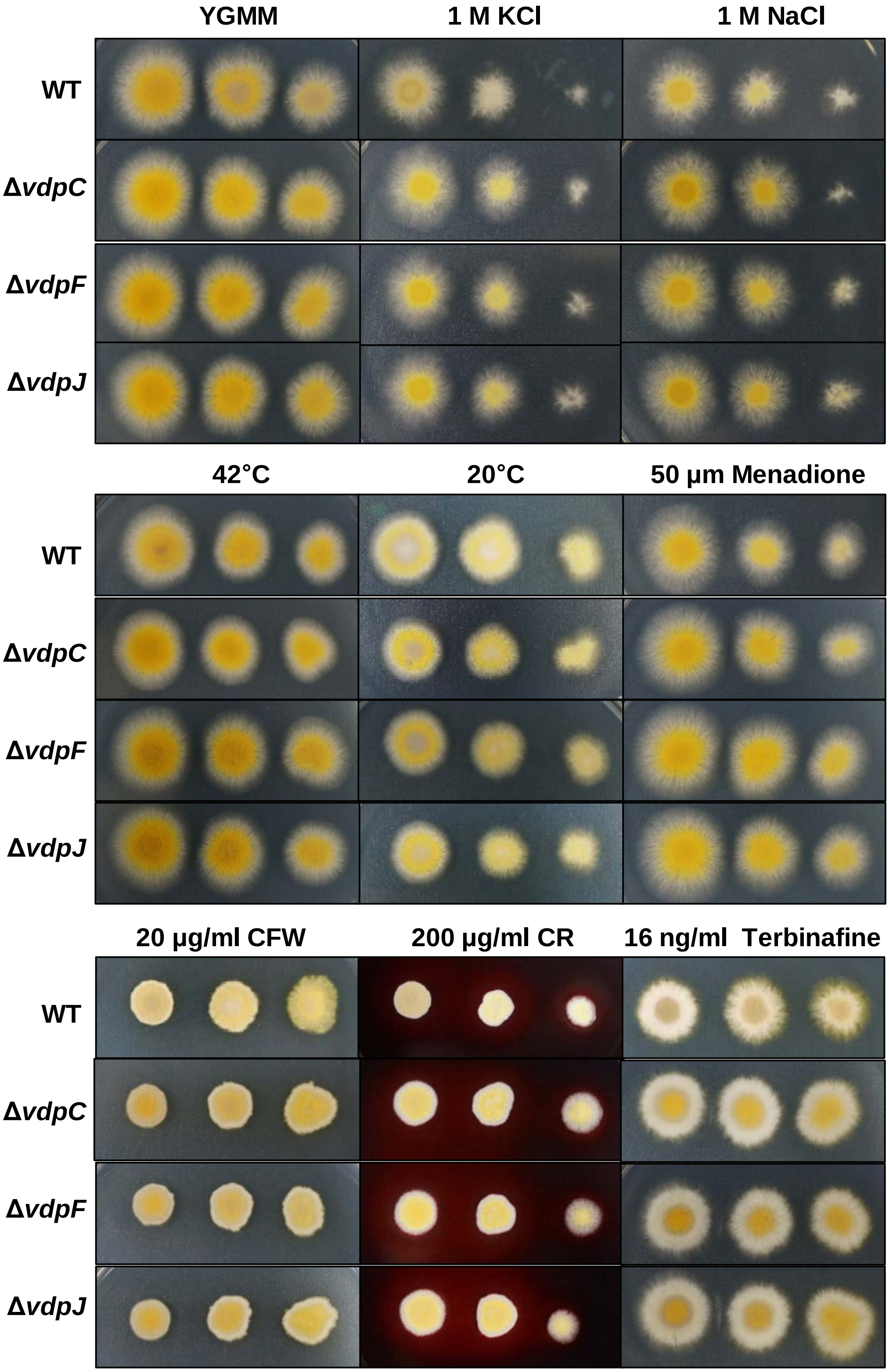
YGMM
1 M KCl
1 M NaCl
WT
ΔvdpC
ΔvdpF
ΔvdpJ
42°C
20°C
50 µm Menadione
20 µg/ml CFW
200 µg/ml CR
16 ng/ml Terbinafine

Supplementary Figure S3. Phenotypes of *vdp*-deletion strains (Δ*vdpC,* Δ*vdpF*, and Δ*vdpJ*).

(A) Colony morphology. Spores were inoculated on GMM or YGMM and incubated for 2 days at 37°C. (B) Spores were inoculated in liquid GMM and incubated for 2 days in 37°C. (C) Asexual reproductive organs. Strains were cultured on coverslips for 24 h on solid CM and observed using a DIC microscope. (D) Sexual reproductive organs. Mycelial balls from liquid cultures were transferred to solid MMCA and incubated under conditions which induce sexual development. Cleistothecia and Hülle cells (top) and ruptured cleistothecia (bottom). SM: Stereomicroscope, DIC: Differential interference contrast. (E) Sensitivity tests to osmotic stress (KCl and NaCl), temperature-stress, oxidative stress (menadione), cell wall- (CFW and CR) and cell membrane-stress (terbinafine). CFW: Calcofluor white, CR: Congo red. Cells with 10-fold serial dilutions (10^4 ,10^3, and 10^2) were spotted onto YGMM containing the stress-imposing agents.

Supplementary Figure S4

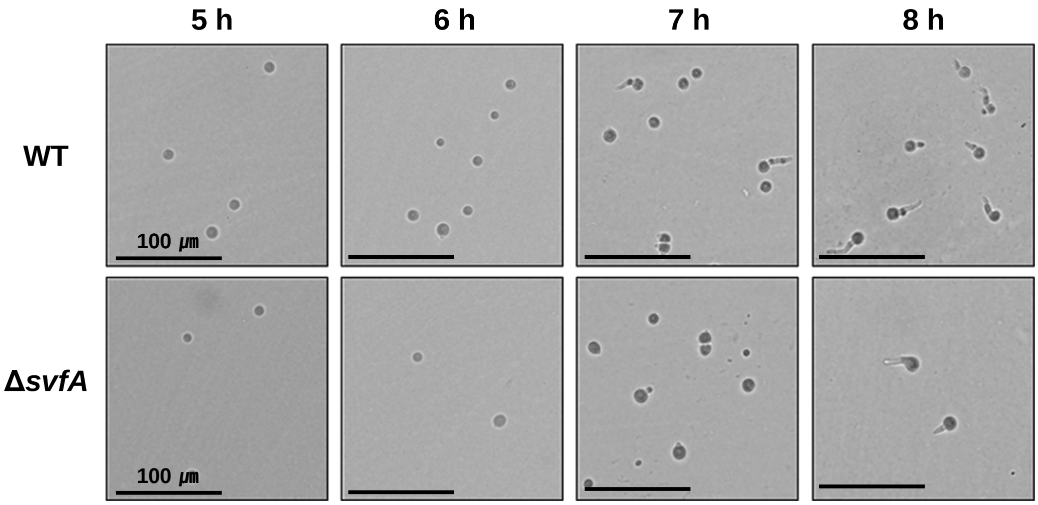

Supplementary Figure S4. Morphology of germ-tube in MM without glucose.

Conidia of WT and Δ*svfA* strain were incubated in liquid MM without glucose at 30°C, and germ-tube formation was observed at indicated time points post-incubation.

Supplementary Figure S5

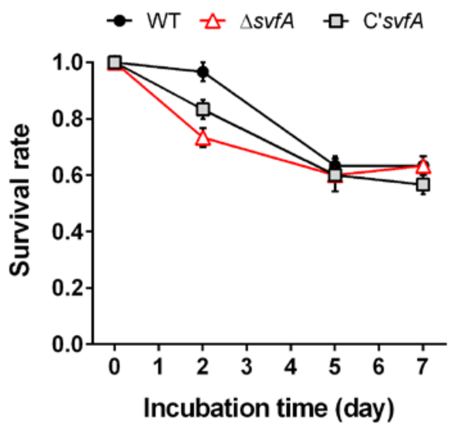

Supplementary Figure S5. Viability of conidia.

Fresh conidia suspension prepared with 2 day old cultures of WT, Δ*svfA*, and C'*svfA* (complementation strain) strains were inoculated on solid GMM and incubated at 37°C. At indicated time points post-incubation, conidia were collected from each cultures and counted. Approximately 200 conidia were spread onto solid GMM, incubated for 2 days at 37°C in triplicates, and number of colonies formed were counted. Survival rates were calculated as a ratio of the number of viable colonies to the number of spores inoculated.

Supplementary Figure S6

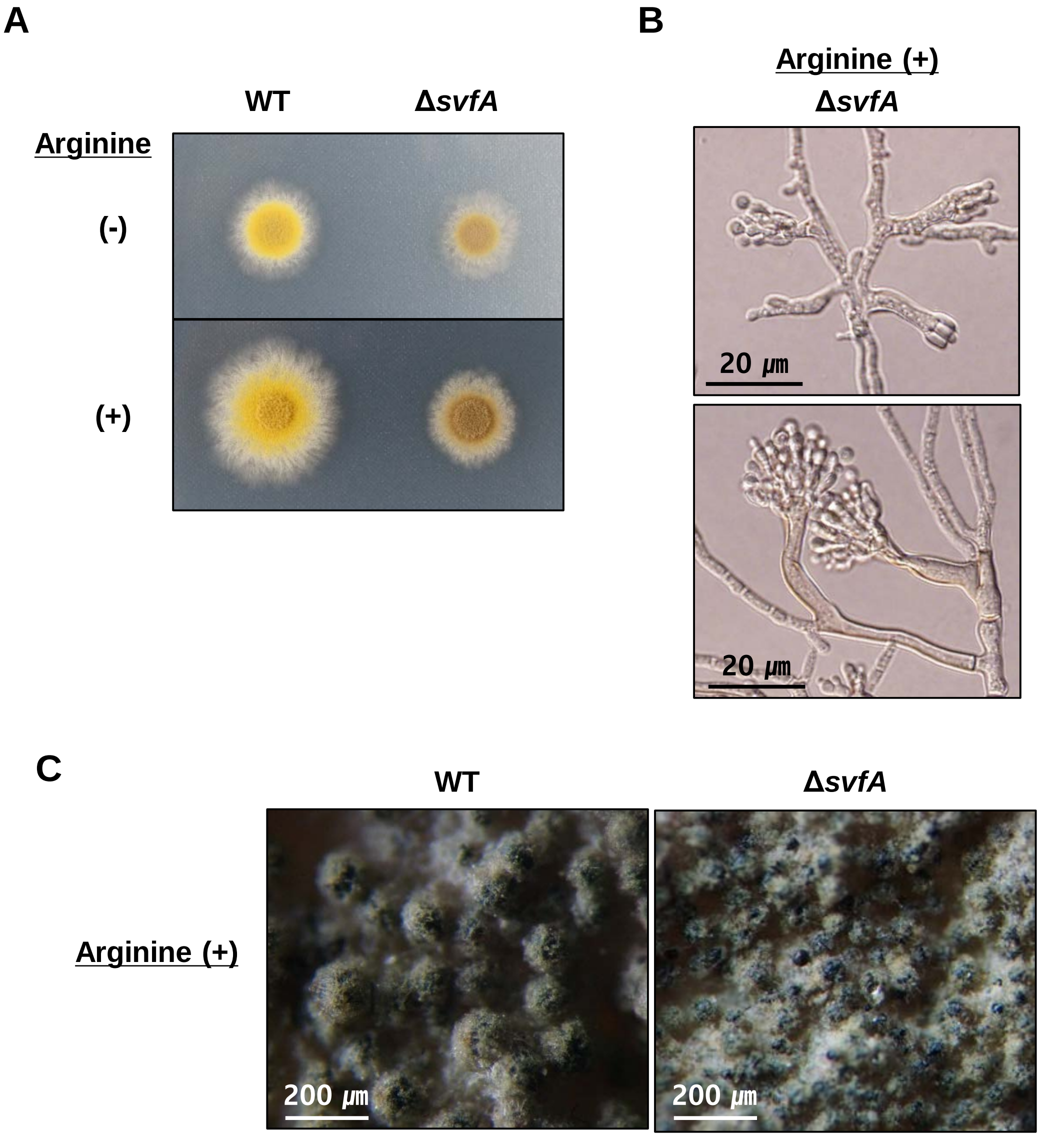

Supplementary Figure S6. Effect of arginine-supplemented medium on Δ*svfA* phenotypes.

(A) Colony morphology. Spores of WT and Δ*svfA* strains were inoculated on YGMM plate with (+) and without (-) arginine and incubated for 2 days at 37°C. (B) Morphology of asexual reproductive organs in Δ*svfA* strain incubated on arginine-containing medium. (C) Small cleistothecia from Δ*svfA* strain incubated on arginine-containing medium.

Supplementary Figure S7

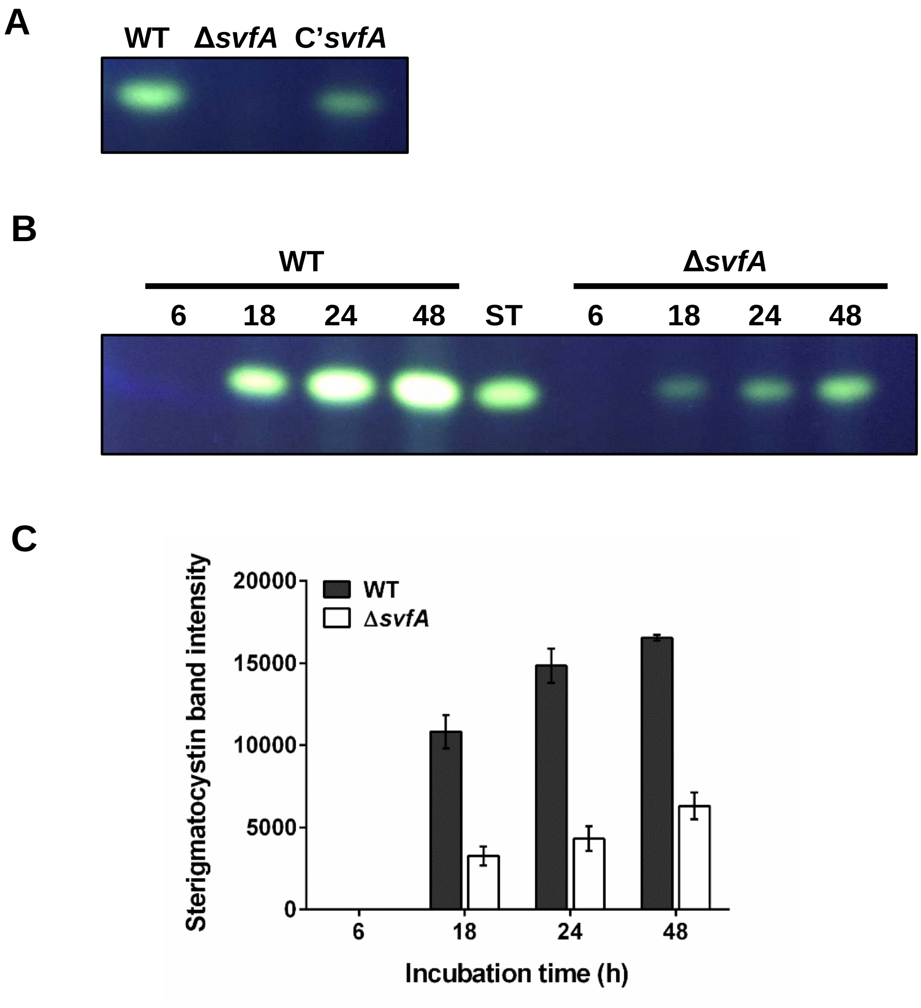

Supplementary Figure S7. TLC analysis of sterigmatocystin (ST).

(A) TLC analysis of ST in the chloroform extracts of WT, Δ*svfA* and C'*svfA* cultures on solid GMM for 4 days. Samples were loaded onto a TLC silica plates and developed in a mobile phase composed of toluene: ethyl acetate: acetic acid (80:10:10 v/v/v). Spots were visualized by aluminum chloride (20% w/v in 95% ethanol) under UV (320 nm). (B) TLC analysis of ST of WT and Δ*svfA*. Equal amount of mycelial balls from 18 h liquid cultures were transferred to solid GMM. After additional incubation (6, 18, 24 and 48 h), mycelia were extracted with chloroform. Samples and ST standard (Sigma-Aldrich) were separated on TLC silica plates and developed. (C) Densitometry displaying relative intensity of the ST bands. The ST bands were normalized to wild-type (6 h). Values are the means of three replicates. The error bar represents standard error of the mean.

Supplementary Figure S8

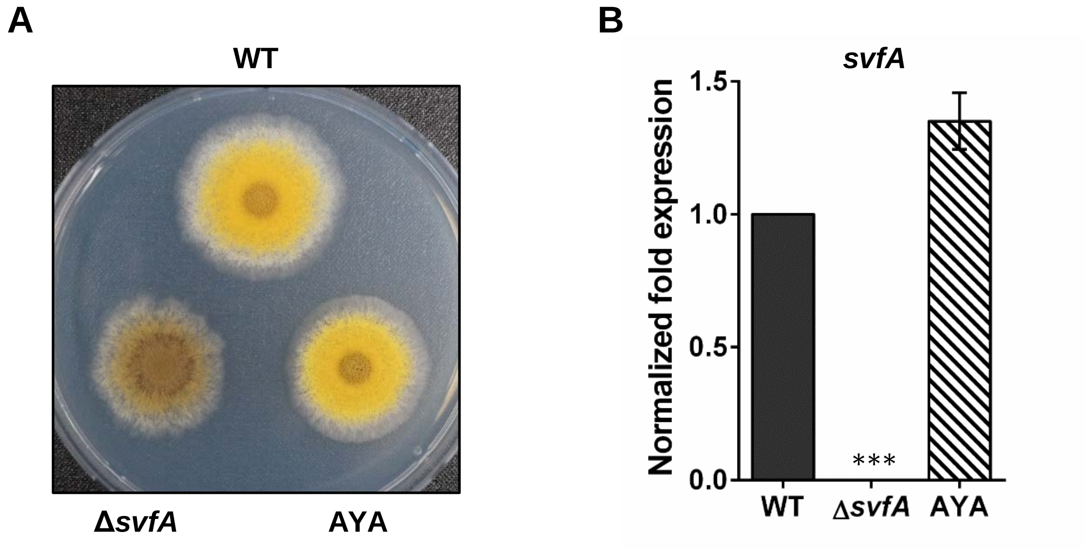

Supplementary Figure S8. Complementation of SvfA::3x YFP strain.

(A) Colony morphology. Spores of WT, Δ*svfA* and SvfA::3x YFP (AYA) strains were inoculated on YGMM plate and incubated for 2 days at 37°C. (B) Expression patterns of *svfA* gene in WT, Δ*svfA*, and AYA strains. Total RNAs extracted from liquid cultures were used for RT-qPCR analyses. 18S rRNA gene served as an internal control. The error bar represents standard error of the mean. ***P < 0.001. No signal was detected in Δ*svfA* strain.

Supplementary Figure S9

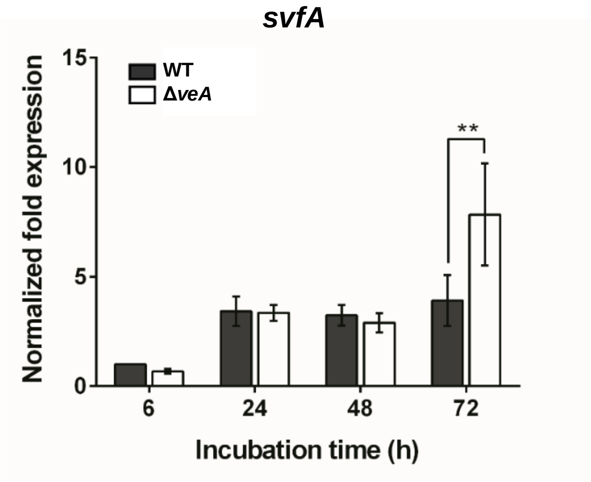

Supplementary Figure S9. Expression patterns of *svfA* gene in WT and Δ*veA* strains.
Total RNAs extracted from sexual development cultures were used for RT-qPCR analyses. 18S rRNA gene served as an internal control. The error bar represents standard error of the mean. ∗∗P < 0.01.

Supplementary Figure S10

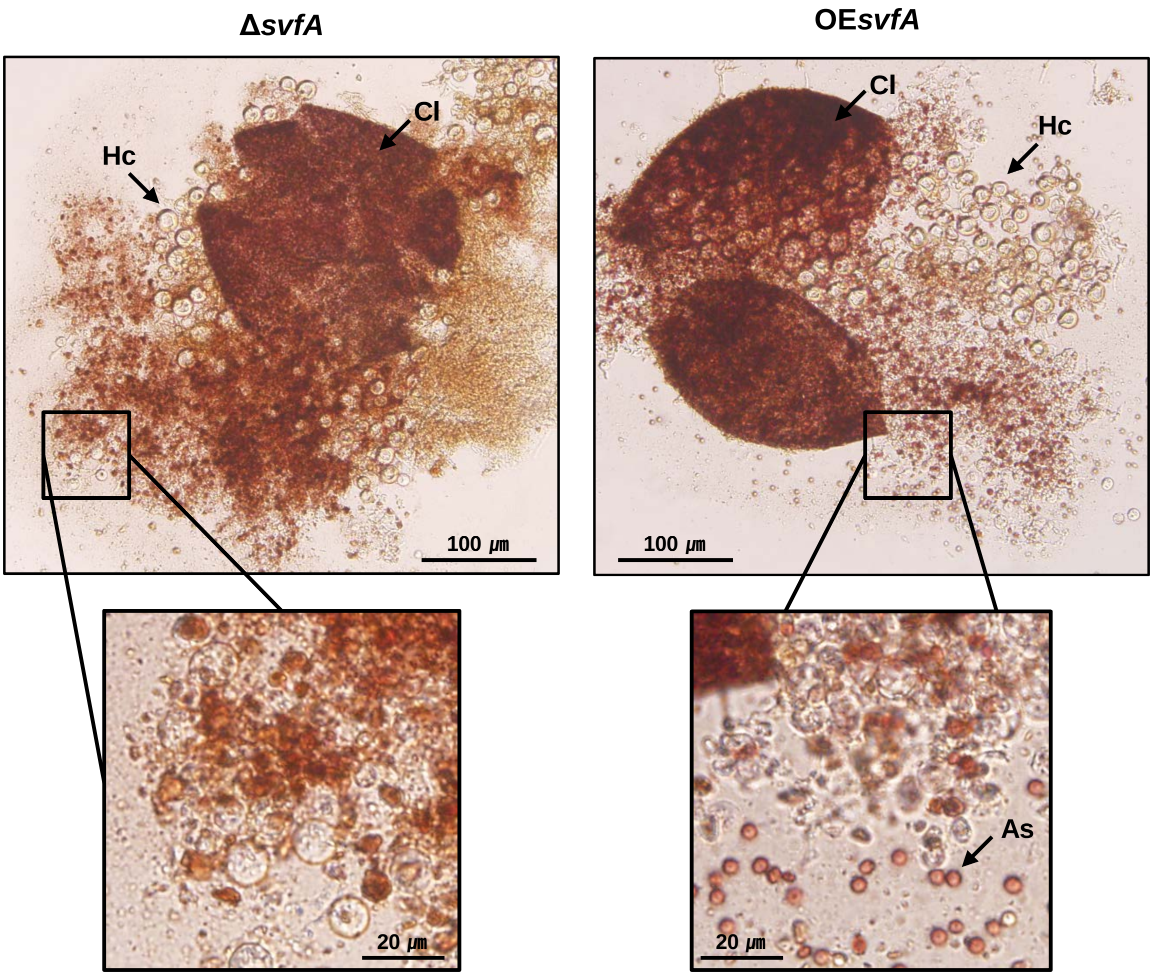

Supplementary Figure S10. Sexual reproductive organs in Δ*svfA* and OE*svfA* strains.

Spores were inoculated on MM3G (MM with 3% glucose) medium containing 0.6% sodium acetate for 6 days. Top panel shows a ruptured cleistothecium, releasing their contents. Globose Hülle cells were seen around the cleistothecia. Bottom panel shows enlarged photographs of square in top panel. Cl: Cleistothecium, Hc: Hülle cells, As: Ascospore

Supplementary Figure S11

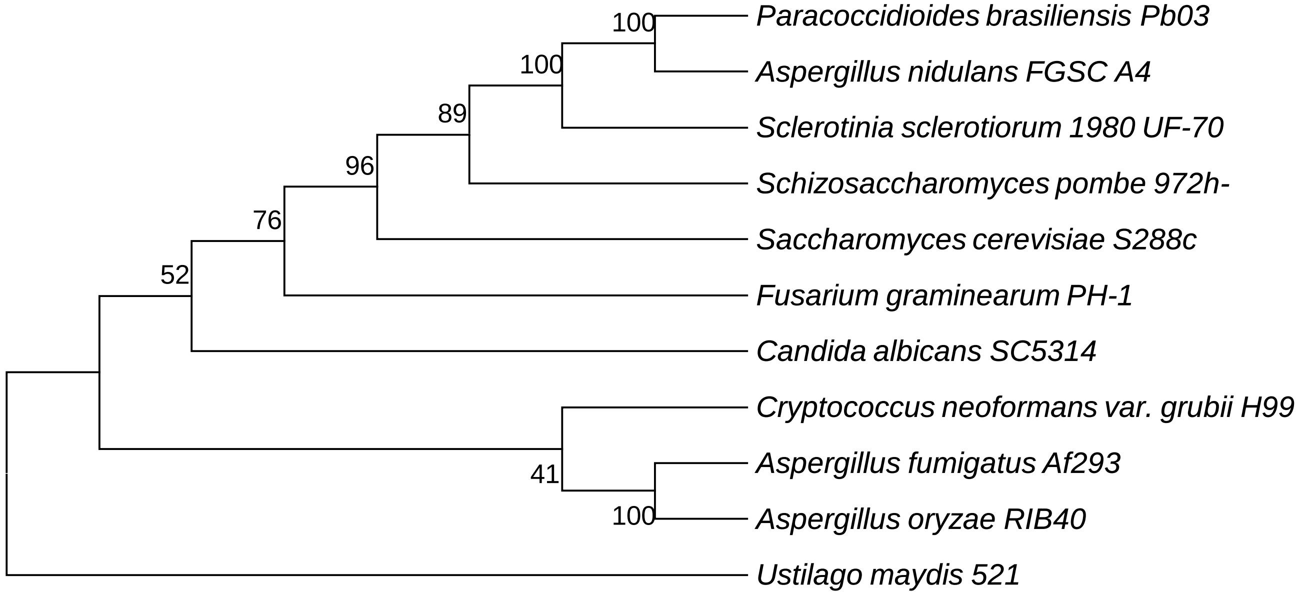

Supplementary Figure S11. The evolutionary relationships of fungi with Svf1 orthologs. Neighbor-joining phylogenetic tree inferred from the amino acid sequence of Svf1 from 11 fungi. Bootstrap values are indicated on branches. Evolutionary analyses were conducted in MEGA7. GenBank accession number of the amino acid sequences retrieved: *Aspergillus fumigatus* (XP_753469.1), *Aspergillus nidulans* (XP_657721.1), *Aspergillus oryzae* (XP_001824034.1), *Candida albicans* (XM_713937.1), *Cryptococcus neoformans* (XM_012191280.1), *Fusarium graminearum* (XM_011325259.1), *Paracoccidiodes brasiliensis* (XM_010762495.1) *Saccharomyces cerevisiae* (CP020126.1), *Schizosaccharomyces pombe* (NM_001023206.2), *Sclerotinia sclerotiorum* (XM_001597673.1), *Ustilago maydis* (XM_011393354.1).

Supplementary Table S1.
***Aspergillus* strains used in this study**

Strains	Genotype	Source
FGSC A4	*veA*$^{+}$	FGSC
KVE9	Δ*veA::argB*	This study
TJ1-1	*yA2; argB2*; *pyroA4; veA*$^{+}$	This study
IsoWT	*yA2; pyroA4*; *veA*$^{+}$	This study
Δ*svfA*	*yA2; argB2*; *pyroA4*; Δ*svfA::argB*; *veA*$^{+}$	This study
Δ*vdpC*	*yA2; argB2*; *pyroA4*; Δ*vdpC::argB*; *veA*$^{+}$	This study
Δ*vdpF*	*yA2; argB2*; *pyroA4*; Δ*vdpF::argB*; *veA*$^{+}$	This study
Δ*vdpJ*	*yA2; argB2*; *pyroA4*; Δ*vdpJ::argB*; *veA*$^{+}$	This study
C'*svfA*	*yA2; argB2*; *pyroA4*; Δ*svfA::argB*; *veA*$^{+}$; *pyroA::svfA::FLAG::trpC(t)*	This study
AYA	*yA2; argB2*; *pyroA4,* Δ*svfA::argB; veA*$^{+}$; *pyroA::svfA::YFP3x::trpC(t)*	This study
VED13A	*pabaA1*; *argB2*; *pyroA4*; *chaA1,* Δ*veA::argB; pyroA::svfA::FLAG::trpC(t)*	This study
Δ*veA28*	*pabaA1*; *argB2*; *pyroA4*; *chaA1,* Δ*veA::argB*	D.M. Han
Δ*veA;* OE*svfA*	*pabaA1*; *argB2*; *pyroA4*; *chaA1,* Δ*veA::argB, pyroA::niiA(p)::svfA::trpC(t)*	This study
Δ*svfA;* OE*svfA*	*yA2*; *argB2*; *pyroA4*; Δ*svfA::argB; veA*$^{+}$, *pyroA::niiA(p)::svfA::trpC(t)*	This study

Supplementary Table S2.
Primers used in this study

	Sequence (5' - 3')
**Double-joint PCR**	
svfA 5for	CGCCGGTATCATAATATCCA
svfA 5rev-argB	AGTCAAATGAGGCCTCTAAACTGGTCACAGGGAAAGTGGAAA
svfA argB-3for	AGCCAAGGTAGATCCAGGCCTAACACATGTTTCCTTTGTAA
svfA 3rev	AGGAAGTGGAGCTCCGACTT
svfA nest for	GGCCTGTGCCATAACACTCT
svfA nest rev	AGCCACACCAACACAAAC
vdpC 5for	AATATGGGCTAGCTCAGCAAA
vdpC 5rev-argB	AGTCAAATGAGGCCTCTAAACTGGTCACTCTGTTGGACCAGGTATTGA
vdpC argB-3for	AGCCAAGGTAGATCCAGGCCTAACACATGGGACTATTGCATTGTAGATAGC
vdpC 3rev	ATAACCCCGAGGATGCG
vdpC nest for	GCTTGTTGACTTGAGTCAGAAA
vdpC nest rev	GCGGCTGAGGCGGCGCGTCA
vdpF 5for	CGGCCATCCCCAAGCTCG
vdpF 5rev-argB	AGTCAAATGAGGCCTCTAAACTGGTCATGTTGCTATTACCCAGGATC
vdpF argB-3for	AGCCAAGGTAGATCCAGGCCTAACACAGGTCTTCCAGTGACCTTGATCA
vdpF 3rev	AAACGCAGGTCTCGCAAG
vdpF nest for	CTGCTGTCCTGTTACTTGGC
vdpF nest rev	AAGACACAGCTTGGATACTGG
vdpJ 5 For	GCCAACGGCCTTACCTTCT
vdpJ 5Rev-argB	AGTCAAATGAGGCCTCTAAACTGGTCATTTGTTGATTTCAGAGAGGT
vdpJ argB-3 For	AGCCAAGGTAGATCCAGGCCTAACACATACCTGAAATTGAGTCATTGCA
vdpJ 3Rev	CTTCCTATTCCATCCGCT
vdpJ nest for	CATTCCACAAGCTGTTGTGG
vdpJ nest rev	GCATCCGCTCAAGACGA
argB for	GACCAGTTTAGAGGCCTC
argB rev	GTGTTAGGCCTGGATCTA
**Southern probe**	
vdpC probe for	GGCGACTCCCATAGTTCTG
vdpC probe rev	TAGGCCGGCAGTCTAA
vdpF probe for	GGGCAAATGCCAGATCC
vdpF probe rev	CGGGCAGTGGTGGATAAA
vdpJ probe for	CCCTTCCTTCGTTCAACA
**qRT-PCR**	
18s qRT F	CTTGGATTTGCTGAAGACTAAC
18s qRT R	CTAACTTTCGTTCCCTGATTAATG
svfA qRT F	GTTCTCTCAGGCTCTTCA
svfA qRT R	GATACCGCCAACGTTAAC
brlA qRT F	TCATATACAGACTTTCCGACCTCTC
brlA qRT R	AAGACCTGATTGGGGTAGTGGGG
abaA qRT F	GACTCATTCCTCAAGGGTG
abaA qRT R	GCATCAGCCTCATGGGTTC

vosA qRT F	ATGGCGGGTTCTTCGTATGG
vosA qRT R	AGCGAGAATTTTAGTCGAAA
mutA qRT F	GAGGGCAGTAGATCATCCAATC
mutA qRT R	TCAATCGATGTCGTCGCGTG
nsdD qRT F	CATCTCACCAGCCACAATTACAGGCG
nsdD qRT R	TTGCGAGCCAGACACAGAGGTCAT
veA qRT F	CCATCTCAAACAACATCGCT
veA qRT R	TGGTGTTTTCGACCTAG
steA qRT F	AATTCGAGGAGGGCATCTTT
steA qRT R	GGTTCTTCGAGCGTAGCATC
esdC qRT F	TCGTTGTCTTCTCGCAGCA
esdC qRT R	AAACTGAAGGCGGATCGGA

Supplementary Table S3.

GO classification of proteins using FungiFun2, according to their biological process

GO ID	GO name	Exact p-value	Adjusted p-value	# genes / category	# genes / input
GO:0097308	cellular response to farnesol	2.5028e-8	0.000002265	7 / 46	7 / 76
GO:0043458	ethanol biosynthetic process	1.2744E-06	0.00009227	3 / 4	3 / 76
GO:0071470	cellular response to osmotic stress	0.000010983	0.00036151	5 / 43	5 / 76
GO:0006083	acetate metabolic process	0.000010985	0.00036151	3 / 7	3 / 76
GO:0033609	oxalate metabolic process	0.000017489	0.00050632	3 / 8	3 / 76
GO:0034599	cellular response to oxidative stress	0.000018183	0.00050632	6 / 80	6 / 76
GO:0007163	establishment or maintenance of cell polarity	0.000025671	0.00066378	5 / 51	5 / 76
GO:0006011	UDP-glucose metabolic process	0.00004743	0.0011447	2 / 2	2 / 76
GO:0010499	proteasomal ubiquitin-independent protein catabolic process	0.00011032	0.0023491	3 / 14	3 / 76
GO:0006012	galactose metabolic process	0.00024242	0.0041788	3 / 18	3 / 76
GO:0006098	pentose-phosphate shunt	0.00024242	0.0041788	3 / 18	3 / 76
GO:0006094	gluconeogenesis	0.00028644	0.0045083	3 / 19	3 / 76
GO:0034605	cellular response to heat	0.00032936	0.0046685	4 / 48	4 / 76
GO:0015976	carbon utilization	0.00033531	0.0046685	3 / 20	3 / 76
GO:0006071	glycerol metabolic process	0.00038925	0.0052188	3 / 21	3 / 76
GO:0005978	glycogen biosynthetic process	0.00046793	0.0058411	2 / 5	2 / 76
GO:0006096	glycolytic process	0.00051318	0.0061923	3 / 23	3 / 76
GO:0043161	proteasome-mediated ubiquitin-dependent protein catabolic process	0.00092594	0.0099253	3 / 28	3 / 76
GO:0044182	filamentous growth of a population of unicellular organisms	0.00092594	0.0099253	3 / 28	3 / 76
GO:0006457	protein folding	0.0013834	0.013911	4 / 70	4 / 76
GO:0042149	cellular response to glucose starvation	0.0016545	0.015357	2 / 9	2 / 76
GO:0051211	anisotropic cell growth	0.0016545	0.015357	2 / 9	2 / 76
GO:0005992	trehalose biosynthetic process	0.0016545	0.015357	2 / 9	2 / 76
GO:0006090	pyruvate metabolic process	0.0020588	0.017745	2 / 10	2 / 76
GO:0006066	alcohol metabolic process	0.0025051	0.021089	2 / 11	2 / 76
GO:0044416	induction by symbiont of host defense response	0.0029926	0.02413	2 / 12	2 / 76
GO:0051666	actin cortical patch localization	0.0040893	0.02413	2 / 14	2 / 76
GO:0055114	oxidation-reduction process	0.0044667	0.02413	12 / 736	12 / 76
GO:0019521	D-gluconate metabolic process	0.0046974	0.02413	2 / 15	2 / 76
GO:0007033	vacuole organization	0.0067534	0.02413	2 / 18	2 / 76
GO:0019615	catechol catabolic process, ortho-cleavage	0.0069324	0.02413	1 / 1	1 / 76
GO:0030835	negative regulation of actin filament depolymerization	0.0069324	0.02413	1 / 1	1 / 76
GO:0030518	intracellular steroid hormone receptor signaling pathway	0.0069324	0.02413	1 / 1	1 / 76
GO:0000060	protein import into nucleus, translocation	0.0069324	0.02413	1 / 1	1 / 76
GO:0019255	glucose 1-phosphate metabolic process	0.0069324	0.02413	1 / 1	1 / 76
GO:0051171	regulation of nitrogen compound metabolic process	0.0069324	0.02413	1 / 1	1 / 76
GO:0006532	aspartate biosynthetic process	0.0069324	0.02413	1 / 1	1 / 76
GO:0006452	translational frameshifting	0.0069324	0.02413	1 / 1	1 / 76
GO:0016119	carotene metabolic process	0.0069324	0.02413	1 / 1	1 / 76
GO:0006085	acetyl-CoA biosynthetic process	0.0069324	0.02413	1 / 1	1 / 76
GO:0019427	acetyl-CoA biosynthetic process from acetate	0.0069324	0.02413	1 / 1	1 / 76
GO:0019593	mannitol biosynthetic process	0.0069324	0.02413	1 / 1	1 / 76
GO:0019307	mannose biosynthetic process	0.0069324	0.02413	1 / 1	1 / 76
GO:0018158	protein oxidation	0.0069324	0.02413	1 / 1	1 / 76
GO:0051784	negative regulation of nuclear division	0.0069324	0.02413	1 / 1	1 / 76
GO:0005991	trehalose metabolic process	0.0069324	0.02413	1 / 1	1 / 76
GO:0042981	regulation of apoptotic process	0.0069324	0.02413	1 / 1	1 / 76

GO:1900435	negative regulation of filamentous growth of a population of unicellular organisms in response to starvation	0.0069324	0.02413	1 / 1	1 / 76
GO:0090475	lys-lys specific dibasic protein processing	0.0069324	0.02413	1 / 1	1 / 76
GO:0042426	choline catabolic process	0.0069324	0.02413	1 / 1	1 / 76
GO:0042183	formate catabolic process	0.0069324	0.02413	1 / 1	1 / 76
GO:0000302	response to reactive oxygen species	0.0069324	0.02413	1 / 1	1 / 76
GO:0046386	deoxyribose phosphate catabolic process	0.0069324	0.02413	1 / 1	1 / 76
GO:0036279	positive regulation of protein export from nucleus in response to glucose starvation	0.0069324	0.02413	1 / 1	1 / 76
GO:2000114	regulation of establishment of cell polarity	0.0069324	0.02413	1 / 1	1 / 76
GO:0002181	cytoplasmic translation	0.0083118	0.028656	2 / 20	2 / 76
GO:0042823	pyridoxal phosphate biosynthetic process	0.013817	0.035728	1 / 2	1 / 76
GO:0042867	pyruvate catabolic process	0.013817	0.035728	1 / 2	1 / 76
GO:0046580	negative regulation of Ras protein signal transduction	0.013817	0.035728	1 / 2	1 / 76
GO:0051764	actin crosslink formation	0.013817	0.035728	1 / 2	1 / 76
GO:0045905	positive regulation of translational termination	0.013817	0.035728	1 / 2	1 / 76
GO:0000947	amino acid catabolic process to alcohol via Ehrlich pathway	0.013817	0.035728	1 / 2	1 / 76
GO:0045337	farnesyl diphosphate biosynthetic process	0.013817	0.035728	1 / 2	1 / 76
GO:0006875	cellular metal ion homeostasis	0.013817	0.035728	1 / 2	1 / 76
GO:0090474	arg-arg specific dibasic protein processing	0.013817	0.035728	1 / 2	1 / 76
GO:0045948	positive regulation of translational initiation	0.013817	0.035728	1 / 2	1 / 76
GO:0001932	regulation of protein phosphorylation	0.013817	0.035728	1 / 2	1 / 76
GO:0051447	negative regulation of meiotic cell cycle	0.013817	0.035728	1 / 2	1 / 76
GO:0005979	regulation of glycogen biosynthetic process	0.013817	0.035728	1 / 2	1 / 76
GO:0080129	proteasome core complex assembly	0.013817	0.035728	1 / 2	1 / 76
GO:0090473	lys-arg specific dibasic protein processing	0.013817	0.035728	1 / 2	1 / 76
GO:0046187	acetaldehyde catabolic process	0.020655	0.043984	1 / 3	1 / 76
GO:0042744	hydrogen peroxide catabolic process	0.020655	0.043984	1 / 3	1 / 76
GO:0008615	pyridoxine biosynthetic process	0.020655	0.043984	1 / 3	1 / 76
GO:0009051	pentose-phosphate shunt, oxidative branch	0.020655	0.043984	1 / 3	1 / 76
GO:0016126	sterol biosynthetic process	0.020655	0.043984	1 / 3	1 / 76
GO:0019379	sulfate assimilation, phosphoadenylyl sulfate reduction by phosphoadenylyl-sulfate reductase (thioredoxin)	0.020655	0.043984	1 / 3	1 / 76
GO:0051083	'de novo' cotranslational protein folding	0.020655	0.043984	1 / 3	1 / 76
GO:0017157	regulation of exocytosis	0.020655	0.043984	1 / 3	1 / 76
GO:0045901	positive regulation of translational elongation	0.020655	0.043984	1 / 3	1 / 76
GO:0045860	positive regulation of protein kinase activity	0.020655	0.043984	1 / 3	1 / 76
GO:0019563	glycerol catabolic process	0.020655	0.043984	1 / 3	1 / 76
GO:0007039	protein catabolic process in the vacuole	0.020655	0.043984	1 / 3	1 / 76
GO:0006401	RNA catabolic process	0.020655	0.043984	1 / 3	1 / 76
GO:1900444	negative regulation of filamentous growth of a population of unicellular organisms in response to biotic stimulus	0.020655	0.043984	1 / 3	1 / 76
GO:0006014	D-ribose metabolic process	0.020655	0.043984	1 / 3	1 / 76
GO:0006078	(1->6)-beta-D-glucan biosynthetic process	0.020655	0.043984	1 / 3	1 / 76
GO:0000492	box C/D snoRNP assembly	0.020655	0.043984	1 / 3	1 / 76
GO:0006521	regulation of cellular amino acid metabolic process	0.020655	0.043984	1 / 3	1 / 76
GO:0016125	sterol metabolic process	0.021798	0.046146	2 / 33	2 / 76
